# Supplementary material for: Stochastic sampling effects favor manual over digital contact tracing
Source: Nat Commun. 2021 Mar 26;12:1919. doi: 10.1038/s41467-021-22082-7 (PMC7997996; doi:10.1038/s41467-021-22082-7)
Supplement: Supplementary file 1 — Supplementary Information [file 41467_2021_22082_MOESM1_ESM.pdf]

Supplementary Information for "Stochastic sampling effects favor manual over digital contact tracing"

Marco Mancastroppa,[1,2] Claudio Castellano,[3] Alessandro Vezzani,[4,1] and Raffaella Burioni[1,2,*]

[1] *Dipartimento di Scienze Matematiche, Fisiche e Informatiche, Università degli Studi di Parma, Parco Area delle Scienze, 7/A 43124 Parma, Italy*
[2] *INFN, Sezione di Milano Bicocca, Gruppo Collegato di Parma, Parco Area delle Scienze, 7/A 43124 Parma, Italy*
[3] *Istituto dei Sistemi Complessi (ISC-CNR), Via dei Taurini 19, I-00185 Roma, Italy*
[4] *Istituto dei Materiali per l'Elettronica ed il Magnetismo (IMEM-CNR), Parco Area delle Scienze, 37/A-43124 Parma, Italy*

Contents

I. Supplementary Method 1: Mean-field equations and analytical derivation of the epidemic thresholds 1
- A. Manual CT 2
- B. Digital CT 5
- C. Hybrid CT 9
- D. Limit cases 11
  - 1. Non-adaptive case (NA) 11
  - 2. Isolation of only symptomatic nodes 11
  - 3. Homogeneous case without delays and without limited scalability 12
  - 4. Heterogeneous case 12

II. Supplementary Method 2: Continuous time Gillespie-like algorithm for network dynamics and epidemic evolution 13

III. Supplementary Notes: Robustness of the results 14
- A. Activity-attractiveness distribution, limited scalability parameters and delays 14
- B. Epidemic active phase 14
- C. Deterministic household CT 14
- D. Correlation between probability of app adoption and individual activity 16

Supplementary References 18

In this supplementary information we derive the mean-field equations for the temporal evolution of the epidemic model on adaptive activity-driven networks. We also derive analytically the epidemic threshold and we describe the scheme of the continuous-time Gillespie-like algorithm used for the numerical approach to the temporal evolution of the network and of the epidemic. Finally, we discuss in detail the robustness of the results, relaxing assumptions in activity distribution, changing most CT parameters in the modelling scheme, introducing further realistic features such as deterministic household CT and correlation between app adoption and individual activity.

I. SUPPLEMENTARY METHOD 1: MEAN-FIELD EQUATIONS AND ANALYTICAL DERIVATION OF THE EPIDEMIC THRESHOLDS

We consider the epidemic model proposed in the main text evolving on an adaptive activity-driven network in the presence of contact tracing of asymptomatic nodes.

The epidemic model proposed is a Susceptible-Infected-Recovered (SIR) model, with a further distinction for the states of infection I. The distinction is based on the presence of symptoms, on tracing and isolation and it models changes in social behaviour depending on nodes' health status. We do not consider here burstiness effects [1] nor memory [2].

*Electronic address: raffaella.burioni@unipr.it

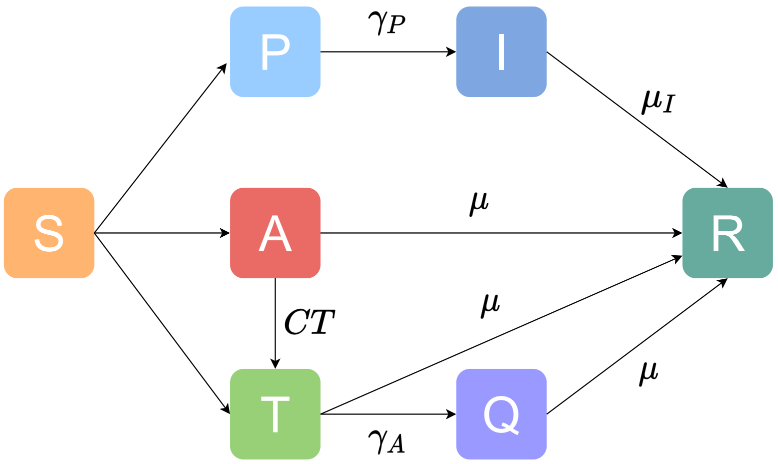

Supplementary Fig. 1: **Epidemic model with contact tracing.** We plot the scheme of the epidemic model with the transitions for CT. The rates for the infection events (emanating from S) and for the CT events are not indicated, as the relevant events, indicated in the text, are complicated.

A. Manual CT

We first focus on the manual CT. In the original epidemic model, individuals can be in states S (susceptible), P (infected presymptomatic), I (infected symptomatic), A (infected asymptomatic) and R (recovered). In the presence of CT, two additional compartments appear: T (asymptomatic traced) and Q (asymptomatic quarantined). The possible transitions among these states are depicted in Supplementary Fig. 1.

There are two types of events related to CT: an asymptomatic individual A becomes traced T when infected by a presymptomatic node P (forward CT) or when she infects a susceptible node that eventually develops symptoms (backward CT). A traced individual remains infectious $(a_T, b_T) = (a_S, b_S)$ until the presymptomatic starts developing symptoms. Only after that moment she enters quarantine $(a_Q, b_Q) = (0, 0)$. For this reason, the rate for the transition $T \to Q$ is $\gamma_A = 1/\tau_A$, with $\tau_A = \tau_P + \tau_C$, where $\tau_P = 1/\gamma_P$ and τ_C is the delay in manual CT.

In the manual case the tracing is effective with probability $\varepsilon(a_S)$, with a_S activity of the index case. The infection and CT transitions are as follows:

$$P + S \xrightarrow{\lambda\delta} P + P \qquad A + S \xrightarrow{\lambda\delta\varepsilon} T + P \qquad T + S \xrightarrow{\lambda\delta\varepsilon} T + P \tag{1}$$

$$P + S \xrightarrow{\lambda(1-\delta)\varepsilon} P + T \qquad A + S \xrightarrow{\lambda(1-\delta)} A + A \qquad T + S \xrightarrow{\lambda(1-\delta)} T + A \tag{2}$$

$$P + S \xrightarrow{\lambda(1-\delta)(1-\varepsilon)} P + A \qquad A + S \xrightarrow{\lambda\delta(1-\varepsilon)} A + P \qquad T + S \xrightarrow{\lambda\delta(1-\varepsilon)} T + P \tag{3}$$

while the spontaneous transitions are:

$$P \xrightarrow{\gamma_P} I \qquad A \xrightarrow{\mu} R \qquad I \xrightarrow{\mu_I} R \tag{4}$$

$$T \xrightarrow{\gamma_A} Q \qquad T \xrightarrow{\mu} R \qquad Q \xrightarrow{\mu} R \tag{5}$$

Notice that in the event $A + S \to T + P$ both individuals change state; in the event $T + S \xrightarrow{\lambda\delta\varepsilon} T + P$ the individual T is traced two times (the second time from the individual she infects while already traced), but this has actually no consequences.

We apply an *activity-attractiveness based mean-field* approach, dividing the population in classes of nodes with same (a_S, b_S) and considering them statistically equivalent. The model is an exact mean-field since local correlations are continuously destroyed due to link reshuffling: thus the epidemic threshold of the SIR and SIS epidemic models are the same [2]. Therefore, to obtain the epidemic threshold we consider the mean-field equations for the SIS version of the model, in which the recovered nodes become susceptible again without gaining immunity. We assign initially to each node the status of symptomatic (with probability δ) or asymptomatic (with probability $1-\delta$), instead of assigning it at the time of infection. This choice is completely equivalent to the epidemic model described and allows us to write the mean-field equations in a simpler way. Thus, at the mean-field level, the epidemic dynamics is described by the probabilities:

- $P_{a_S,b_S}(t)$ for a symptomatic node to be infected pre-symptomatic at time t;
- $I_{a_S,b_S}(t)$ for a symptomatic node to be infected symptomatic at time t;
- $1 - I_{a_S,b_S}(t) - P_{a_S,b_S}(t)$ to be susceptible at time t, for a node which will develop symptoms;

- $A_{a_S,b_S}(t)$ for an asymptomatic node to be infected asymptomatic at time t;
- $T_{a_S,b_S}(t)$ for an asymptomatic node to be infected traced at time t;
- $Q_{a_S,b_S}(t)$ for an asymptomatic node to be infected isolated at time t;
- $1-A_{a_S,b_S}(t)-Q_{a_S,b_S}(t)-T_{a_S,b_S}(t)$ to be susceptible at time t, for a node which will not develop symptoms.

In this case the average attractiveness at time t is $\langle b(t)\rangle = \overline{b_S} - (1-\delta)\overline{b_S Q}(t) - \delta\overline{b_S I}(t)$ where we define in general $\overline{g} = \int da_S db_S \rho(a_S, b_S) g_{a_S,b_S}$. We consider the system in the thermodynamic limit. The probabilities previously introduced evolve accordingly to the following equations:

$$\begin{aligned}\partial_t P_{a_S,b_S}(t) = -\gamma_P P_{a_S,b_S}(t) + \lambda a_S(1 - I_{a_S,b_S}(t) - P_{a_S,b_S}(t))\frac{\delta\overline{b_S P}(t) + (1-\delta)[\overline{b_S T}(t) + \overline{b_S A}(t)]}{\overline{b_S} - (1-\delta)\overline{b_S Q}(t) - \delta\overline{b_S I}(t)} \\ + \lambda b_S(1 - I_{a_S,b_S}(t) - P_{a_S,b_S}(t))\frac{\delta\overline{a_S P}(t) + (1-\delta)[\overline{a_S T}(t) + \overline{a_S A}(t)]}{\overline{b_S} - (1-\delta)\overline{b_S Q}(t) - \delta\overline{b_S I}(t)}\end{aligned} \tag{6}$$

where the first term on right hand side accounts for symptoms onset; the second and third terms account for contagion processes of a susceptible node who engages a contact with a pre-symptomatic or a non-isolated asymptomatic infected node, respectively for the activation of the susceptible and of the infected node. Both terms are averaged over all the activity-attractiveness classes of the infected node.

$$\partial_t I_{a_S,b_S}(t) = -\mu_I I_{a_S,b_S}(t) + \gamma_P P_{a_S,b_S}(t) \tag{7}$$

where the first term on the right hand side accounts for spontaneous recovery and the second term for spontaneous symptoms development.

$$\begin{aligned}\partial_t A_{a_S,b_S}(t) = -\mu A_{a_S,b_S}(t) + \lambda a_S(1 - A_{a_S,b_S}(t) - T_{a_S,b_S}(t) - Q_{a_S,b_S}(t))\frac{\delta[\overline{b_S P}(t) - \overline{\varepsilon b_S P}(t)] + (1-\delta)[\overline{b_S T}(t) + \overline{b_S A}(t)]}{\overline{b_S} - (1-\delta)\overline{b_S Q}(t) - \delta\overline{b_S I}(t)} \\ + \lambda b_S(1 - A_{a_S,b_S}(t) - T_{a_S,b_S}(t) - Q_{a_S,b_S}(t))\frac{\delta[\overline{a_S P}(t) - \overline{\varepsilon a_S P}(t)] + (1-\delta)[\overline{a_S T}(t) + \overline{a_S A}(t)]}{\overline{b_S} - (1-\delta)\overline{b_S Q}(t) - \delta\overline{b_S I}(t)} \\ - \lambda a_S \delta A_{a_S,b_S}(t)\frac{\overline{\varepsilon b_S} - \overline{\varepsilon b_S I}(t) - \overline{\varepsilon b_S P}(t)}{\overline{b_S} - (1-\delta)\overline{b_S Q}(t) - \delta\overline{b_S I}(t)} - \lambda b_S \delta A_{a_S,b_S}(t)\frac{\overline{\varepsilon a_S} - \overline{\varepsilon a_S I}(t) - \overline{\varepsilon a_S P}(t)}{\overline{b_S} - (1-\delta)\overline{b_S Q}(t) - \delta\overline{b_S I}(t)}\end{aligned} \tag{8}$$

where the first term on right hand side accounts for spontaneous recovery; the second and third terms account for contagion processes of a susceptible node who engages a contact with a non-isolated asymptomatic infected node or with a pre-symptomatic node and their contact is not traced ($(1-\varepsilon(a'_S))$, with a'_S activity of the pre-symptomatic node). Both terms are averaged over all the activity-attractiveness classes of the infected node. The fourth and fifth terms correspond to contact tracing of infected asymptomatic due to infection of a susceptible symptomatic and effective CT of the link ($\varepsilon(a'_S)$, with a'_S activity of the pre-symptomatic node). Both terms are averaged over all the activity-attractiveness classes of the susceptible node.

$$\begin{aligned}\partial_t T_{a_S,b_S}(t) = -(\mu + \gamma_A)T_{a_S,b_S}(t) + \lambda b_S(1 - A_{a_S,b_S}(t) - T_{a_S,b_S}(t) - Q_{a_S,b_S}(t))\frac{\delta\overline{\varepsilon a_S P}(t)}{\overline{b_S} - (1-\delta)\overline{b_S Q}(t) - \delta\overline{b_S I}(t)} \\ + \lambda a_S(1 - A_{a_S,b_S}(t) - T_{a_S,b_S}(t) - Q_{a_S,b_S}(t))\frac{\delta\overline{\varepsilon b_S P}(t)}{\overline{b_S} - (1-\delta)\overline{b_S Q}(t) - \delta\overline{b_S I}(t)} \\ + \lambda b_S \delta A_{a_S,b_S}(t)\frac{\overline{\varepsilon a_S} - \overline{\varepsilon a_S I}(t) - \overline{\varepsilon a_S P}(t)}{\overline{b_S} - (1-\delta)\overline{b_S Q}(t) - \delta\overline{b_S I}(t)} \\ + \lambda a_S \delta A_{a_S,b_S}(t)\frac{\overline{\varepsilon b_S} - \overline{\varepsilon b_S I}(t) - \overline{\varepsilon b_S P}(t)}{\overline{b_S} - (1-\delta)\overline{b_S Q}(t) - \delta\overline{b_S I}(t)}\end{aligned} \tag{9}$$

where the first term on right hand side accounts for isolation and recovery. The second and third terms account for contagion processes of a susceptible node who engage a contact with a pre-symptomatic node and their contact is traced ($\varepsilon(a'_S)$, with a'_S activity of the pre-symptomatic node). Both terms are averaged over all the activity-attractiveness classes of the infected node. The fourth and fifth terms correspond to contact tracing of infected asymptomatic due to infection of a susceptible symptomatic and effective CT of the link ($\varepsilon(a'_S)$, with a'_S activity of the pre-symptomatic node). Both terms are averaged over all the activity-attractiveness classes of the susceptible node.

$$\partial_t Q_{a_S,b_S}(t) = -\mu Q_{a_S,b_S}(t) + \gamma_A T_{a_S,b_S}(t) \tag{10}$$

where the first term on right hand side accounts for spontaneous recovery and the second term for isolation of traced asymptomatic infected nodes.

This set of equations admits as a stationary state the absorbing state, a configuration where all the population is susceptible. To obtain the condition for the stability of the absorbing state, i.e. the epidemic threshold, we apply a linear stability analysis around the absorbing state.

Let us now consider the case of realistic correlations between the activity and attractiveness [3, 4]: $\rho(a_S, b_S) = \rho_S(a_S)\delta(b_S - a_S)$, with generic $\rho_S(a_S)$. If we average the equations on all activity classes, we obtain the temporal evolution of the average probabilities $\overline{P}(t)$, $\overline{I}(t)$, $\overline{A}(t)$, $\overline{T}(t)$, $\overline{Q}(t)$; similarly we obtain the temporal evolution of $\overline{a_S T}(t)$, $\overline{a_S A}(t)$, $\overline{a_S P}(t)$ and $\overline{\varepsilon a_S P}(t)$ multiplying the equations for $a_S\rho_S(a_S)$ or $\varepsilon(a_S)a_S\rho_S(a_S)$ and integrating. Neglecting second order terms in probabilities, we obtain a linearized set of 9 differential equations:

$$\partial_t \overline{I}(t) = -\mu_I \overline{I}(t) + \gamma_P \overline{P}(t) \tag{11}$$

$$\partial_t \overline{P}(t) = -\gamma_P \overline{P}(t) + 2\lambda[\delta \overline{a_S P}(t) + (1-\delta)(\overline{a_S T}(t) + \overline{a_S A}(t))] \tag{12}$$

$$\partial_t \overline{Q}(t) = -\mu \overline{Q}(t) + \gamma_A \overline{T}(t) \tag{13}$$

$$\partial_t \overline{T}(t) = -(\mu + \gamma_A)\overline{T}(t) + 2\lambda\delta\overline{\varepsilon a_S P}(t) + 2\lambda\delta\overline{a_S A}(t)\frac{\overline{\varepsilon a_S}}{\overline{a_S}} \tag{14}$$

$$\partial_t \overline{A}(t) = -\mu\overline{A}(t) + 2\lambda[\delta(\overline{a_S P}(t) - \overline{\varepsilon a_S P}(t)) + (1-\delta)(\overline{a_S T}(t) + \overline{a_S A}(t))] - 2\lambda\delta\overline{a_S A}(t)\frac{\overline{\varepsilon a_S}}{\overline{a_S}} \tag{15}$$

$$\partial_t \overline{a_S P}(t) = -\gamma_P \overline{a_S P}(t) + 2\lambda\frac{\overline{a_S^2}}{\overline{a_S}}[\delta\overline{a_S P}(t) + (1-\delta)(\overline{a_S T}(t) + \overline{a_S A}(t))] \tag{16}$$

$$\partial_t \overline{\varepsilon a_S P}(t) = -\gamma_P \overline{\varepsilon a_S P}(t) + 2\lambda\frac{\overline{\varepsilon a_S^2}}{\overline{a_S}}[\delta\overline{a_S P}(t) + (1-\delta)(\overline{a_S T}(t) + \overline{a_S A}(t))] \tag{17}$$

$$\partial_t \overline{a_S T}(t) = -(\mu + \gamma_A)\overline{a_S T}(t) + 2\lambda\delta\frac{\overline{a_S^2}}{\overline{a_S}}\overline{\varepsilon a_S P}(t) + 2\lambda\delta\overline{a_S^2 A}(t)\frac{\overline{\varepsilon a_S}}{\overline{a_S}} \tag{18}$$

$$\partial_t \overline{a_S A}(t) = -\mu\overline{a_S A}(t) + 2\lambda\frac{\overline{a_S^2}}{\overline{a_S}}[\delta(\overline{a_S P}(t) - \overline{\varepsilon a_S P}(t)) + (1-\delta)(\overline{a_S T}(t) + \overline{a_S A}(t))] - 2\lambda\delta\overline{a_S^2 A}(t)\frac{\overline{\varepsilon a_S}}{\overline{a_S}} \tag{19}$$

The linearized equations for the dynamic evolution of $\overline{a_S^n A}(t)$ and $\overline{a_S^n T}(t)$ always involve terms like $\overline{a_S^{n+1} A}(t)$, due to the contact tracing terms. This would produce an infinite set of coupled linear differential equations: to close the equations and obtain a complete set of linearized equations, we express $\overline{a_S^2 A}(t)$ in terms of the other average probabilities. By definition $\overline{a_S^2 A}(t) = \int da_S \rho_S(a_S) a_S^2 A_{a_S}(t)$: since we are interested in studying the absorbing steady state, we consider Eq. (8) for $\rho(a_S, b_S) = \rho_S(a_S)\delta(b_S - a_S)$, linearized around the absorbing state and near the stationary condition $\partial_t A_{a_S}(t) \sim 0$:

$$A_{a_S}(t) \simeq \frac{2\lambda a_S[\delta(\overline{a_S P}(t) - \overline{\varepsilon a_S P}(t)) + (1-\delta)(\overline{a_S T}(t) + \overline{a_S A}(t))]}{\mu\overline{a_S} + 2\lambda\delta a_S\overline{\varepsilon a_S}} \tag{20}$$

Thus, setting $r = \lambda/\mu$ and replacing Eq. (20) into the definition of $\overline{a_S^2 A}(t)$ we obtain:

$$\overline{a_S^2 A}(t) \simeq \frac{2r}{\overline{a_S}}[\delta(\overline{a_S P}(t) - \overline{\varepsilon a_S P}(t)) + (1-\delta)(\overline{a_S T}(t) + \overline{a_S A}(t))]K \tag{21}$$

where $K = \overline{\frac{a_S^3}{1+2r\delta a_S\frac{\overline{\varepsilon a_S}}{\overline{a_S}}}} = \int da_S \rho_S(a_S)\frac{a_S^3}{1+2r\delta a_S\frac{\overline{\varepsilon a_S}}{\overline{a_S}}}$.

In this way we obtain the following linearized equations for $\overline{a_S T}(t)$ and $\overline{a_S A}(t)$, near the absorbing stationary state:

$$\partial_t \overline{a_S T}(t) = -(\mu + \gamma_A)\overline{a_S T}(t) + 2\lambda\delta\frac{\overline{a_S^2}}{\overline{a_S}}\overline{\varepsilon a_S P}(t) + 4\lambda r\frac{\overline{\varepsilon a_S}}{\overline{a_S}^2}\delta K[\delta(\overline{a_S P}(t) - \overline{\varepsilon a_S P}(t)) + (1-\delta)(\overline{a_S T}(t) + \overline{a_S A}(t))] \tag{22}$$

$$\begin{aligned}\partial_t \overline{a_S A}(t) = -\mu\overline{a_S A}(t) &+ 2\lambda\frac{\overline{a_S^2}}{\overline{a_S}}[\delta(\overline{a_S P}(t) - \overline{\varepsilon a_S P}(t)) + (1-\delta)(\overline{a_S T}(t) + \overline{a_S A}(t))] \\ &- 4\lambda r\frac{\overline{\varepsilon a_S}}{\overline{a_S}^2}\delta K[\delta(\overline{a_S P}(t) - \overline{\varepsilon a_S P}(t)) + (1-\delta)(\overline{a_S T}(t) + \overline{a_S A}(t))]\end{aligned} \tag{23}$$

We focus on the Jacobian matrix of this set of 9 linearized equations:

$$J = \begin{bmatrix} -\mu_I & \gamma_P & 0 & 0 & 0 & 0 & 0 & 0 & 0 \\ 0 & -\gamma_P & 0 & 0 & 0 & 2\lambda\delta & 0 & 2\lambda(1-\delta) & 2\lambda(1-\delta) \\ 0 & 0 & -\mu & \gamma_A & 0 & 0 & 0 & 0 & 0 \\ 0 & 0 & 0 & -\mu-\gamma_A & 0 & 0 & 2\lambda\delta & 0 & 2\lambda\delta\frac{\overline{\varepsilon a_S}}{\overline{a_S}} \\ 0 & 0 & 0 & 0 & -\mu & 2\lambda\delta & -2\lambda\delta & 2\lambda(1-\delta) & 2\lambda(1-\delta)-2\lambda\delta\frac{\overline{\varepsilon a_S}}{\overline{a_S}} \\ 0 & 0 & 0 & 0 & 0 & -\gamma_P+\Delta & 0 & \Gamma & \Gamma \\ 0 & 0 & 0 & 0 & 0 & \phi & -\gamma_P & \Phi & \Phi \\ 0 & 0 & 0 & 0 & 0 & \theta & \Delta-\theta & -\mu-\gamma_A+\Psi & \Psi \\ 0 & 0 & 0 & 0 & 0 & \Delta-\theta & \theta-\Delta & \Gamma-\Psi & -\mu+\Gamma-\Psi \end{bmatrix} = \begin{bmatrix} \mathbb{A}(5x5) & \mathbb{C}(5x4) \\ \mathbb{O}(4x5) & \mathbb{B}(4x4) \end{bmatrix} \tag{24}$$

where $\Delta = 2\lambda\delta\frac{\overline{a_S^2}}{\overline{a_S}}$, $\Gamma = 2\lambda(1-\delta)\frac{\overline{a_S^2}}{\overline{a_S}}$, $\phi = 2\lambda\delta\frac{\overline{\varepsilon a_S^2}}{\overline{a_S}}$, $\Phi = 2\lambda(1-\delta)\frac{\overline{\varepsilon a_S^2}}{\overline{a_S}}$, $\theta = 4\lambda r\delta^2\frac{\overline{\varepsilon a_S}}{\overline{a_S}^2}K$ and $\Psi = 4\lambda r\delta(1-\delta)\frac{\overline{\varepsilon a_S}}{\overline{a_S}^2}K$.

The Jacobian matrix is a block matrix and the condition for the stability of the absorbing state is obtained imposing all eigenvalues to be negative. We can consider separately the two blocks on the diagonal: for the first block $\mathbb{A}$ it is evident that the eigenvalues are $\xi_{1,2} = -\mu$, $\xi_3 = -\mu_I$, $\xi_4 = -\gamma_P$, $\xi_5 = -\mu-\gamma_A$, all negative. Therefore, it is sufficient to study block $\mathbb{B}$, which is a matrix 4x4. The characteristic polynomial of $\mathbb{B}$ is a polynomial of degree 4, thus we apply the Descartes' rule of signs to impose all roots to be negative and we obtain the condition for the stability of the absorbing state:

$$8r^3\delta^2(1-\delta)\frac{\overline{\varepsilon a_S^2}\,\overline{\varepsilon a_S}}{\overline{a_S}}\overline{\frac{a_S^3}{1+2r\delta a_S\frac{\overline{\varepsilon a_S}}{\overline{a_S}}}}\frac{\gamma_A}{\mu} - 4r^2\delta(1-\delta)\left[\overline{\varepsilon a_S^2}\,\overline{a_S^2} + \frac{\gamma_P}{\mu}\overline{\varepsilon a_S}\overline{\frac{a_S^3}{1+2r\delta a_S\frac{\overline{\varepsilon a_S}}{\overline{a_S}}}}\right]\frac{\gamma_A}{\mu} \\ + 2r\overline{a_S^2}\,\overline{a_S}\left(\frac{\gamma_A}{\mu}+1\right)\left(\delta+\frac{\gamma_P}{\mu}(1-\delta)\right) - \overline{a_S}^2\frac{\gamma_P}{\mu}\left(\frac{\gamma_A}{\mu}+1\right) < 0 \tag{25}$$

By setting the equality, the equation allows to obtain a closed relation for estimating the epidemic threshold r_C. The epidemic threshold obtained with the mean-field approach is exact and it holds for manual contact tracing, with arbitrary delay τ_C (encapsulated in γ_A), for arbitrary $\rho_S(a_S)$ and $\varepsilon(a_S)$.

B. Digital CT

We now focus on the digital CT. Each individual is either endowed or not with the app in the initial condition (with a probability $f(a_S)$ depending on her activity). This implies that there are different compartments for individuals without the app (S, I, P, etc.) and with the app (denoted with the superscript $^\alpha$: S^α, I^α, P^α, etc.). Notice that, for pure digital CT, necessarily $T = 0$ and $Q = 0$. We nevertheless write here the transitions involving them, that may play a role for hybrid protocols.

The spontaneous transitions do not depend on whether the individual has the app or not, but the difference with respect to the manual protocol is that $\tau_C = 0$ so that $\gamma_A = \gamma_P$. The infection events are instead as follows. Those involving both individuals without the app (in such a case no new traced individual is generated):

$$P + S \xrightarrow{\lambda\delta} P + P \qquad A + S \xrightarrow{\lambda\delta} A + P \qquad T + S \xrightarrow{\lambda\delta} T + P \tag{26}$$

$$P + S \xrightarrow{\lambda(1-\delta)} P + A \qquad A + S \xrightarrow{\lambda(1-\delta)} A + A \qquad T + S \xrightarrow{\lambda(1-\delta)} T + A \tag{27}$$

Those involving only one individual with the app (in such a case no new traced individual is generated):

$$P^\alpha + S \xrightarrow{\lambda\delta} P^\alpha + P \qquad A^\alpha + S \xrightarrow{\lambda\delta} A^\alpha + P \qquad T^\alpha + S \xrightarrow{\lambda\delta} T^\alpha + P \tag{28}$$

$$P^\alpha + S \xrightarrow{\lambda(1-\delta)} P^\alpha + A \qquad A^\alpha + S \xrightarrow{\lambda(1-\delta)} A^\alpha + A \qquad T^\alpha + S \xrightarrow{\lambda(1-\delta)} T^\alpha + A \tag{29}$$

$$P + S^\alpha \xrightarrow{\lambda\delta} P + P^\alpha \qquad A + S^\alpha \xrightarrow{\lambda\delta} A + P^\alpha \qquad T + S^\alpha \xrightarrow{\lambda\delta} T + P^\alpha \tag{30}$$

$$P + S^\alpha \xrightarrow{\lambda(1-\delta)} P + A^\alpha \qquad A + S^\alpha \xrightarrow{\lambda(1-\delta)} A + A^\alpha \qquad T + S^\alpha \xrightarrow{\lambda(1-\delta)} T + A^\alpha \tag{31}$$

Those involving both individuals with the app (in such a case new traced individuals can be generated):

$$P^\alpha + S^\alpha \xrightarrow{\lambda\delta} P^\alpha + P^\alpha \qquad A^\alpha + S^\alpha \xrightarrow{\lambda\delta} T^\alpha + P^\alpha \qquad T^\alpha + S^\alpha \xrightarrow{\lambda\delta} T^\alpha + P^\alpha \tag{32}$$

$$P^\alpha + S^\alpha \xrightarrow{\lambda(1-\delta)} P^\alpha + T^\alpha \qquad A^\alpha + S^\alpha \xrightarrow{\lambda(1-\delta)} A^\alpha + A^\alpha \qquad T^\alpha + S^\alpha \xrightarrow{\lambda(1-\delta)} T^\alpha + A^\alpha \tag{33}$$

Analogously to the manual CT we apply the *activity-attractiveness based mean-field* approach to the app-based CT. At the mean-field level, the epidemic dynamics is described by the probabilities:

- $P_{a_S,b_S}(t)$ for a symptomatic node without app to be infected pre-symptomatic at time t;
- $I_{a_S,b_S}(t)$ for a symptomatic node without app to be infected symptomatic at time t;
- $1 - P_{a_S,b_S}(t) - I_{a_S,b_S}(t)$ to be susceptible at time t, for a node without app and which will develop symptoms;
- $A_{a_S,b_S}(t)$ for an asymptomatic node without app to be infected asymptomatic at time t;
- $1 - A_{a_S,b_S}(t)$ to be susceptible at time t, for a node without app and which will not develop symptoms;
- $P^{\alpha}_{a_S,b_S}(t)$ for a symptomatic node with app to be infected pre-symptomatic at time t;
- $I^{\alpha}_{a_S,b_S}(t)$ for a symptomatic node with app to be infected symptomatic at time t;
- $1 - P^{\alpha}_{a_S,b_S}(t) - I^{\alpha}_{a_S,b_S}(t)$ to be susceptible at time t, for a node with app and which will develop symptoms;
- $A^{\alpha}_{a_S,b_S}(t)$ for an asymptomatic node with app to be infected asymptomatic at time t;
- $T^{\alpha}_{a_S,b_S}(t)$ for an asymptomatic node with app to be infected traced at time t;
- $Q^{\alpha}_{a_S,b_S}(t)$ for an asymptomatic node with app to be infected isolated at time t;
- $1 - A^{\alpha}_{a_S,b_S}(t) - Q^{\alpha}_{a_S,b_S}(t) - T^{\alpha}_{a_S,b_S}(t)$ to be susceptible at time t, for a node with app and which will not develop symptoms.

In this case the average attractiveness at time t is $\langle b(t)\rangle = \overline{b_S} - (1-\delta)\overline{fb_S Q^{\alpha}}(t) - \delta(\overline{fb_S I^{\alpha}}(t) + \overline{(1-f)b_S I}(t))$. We consider the system in the thermodynamic limit: the probabilities previously introduced evolve according to the following equations, obtained analogously to those for manual CT:

$$\partial_t I_{a_S,b_S}(t) = -\mu_I I_{a_S,b_S}(t) + \gamma_P P_{a_S,b_S}(t) \tag{34}$$

$$\begin{aligned}\partial_t P_{a_S,b_S}(t) = & -\gamma_P P_{a_S,b_S}(t)\\ & + \lambda a_S(1 - I_{a_S,b_S}(t) - P_{a_S,b_S}(t))\frac{\delta(\overline{fb_S P^{\alpha}}(t) + \overline{(1-f)b_S P}(t)) + (1-\delta)(\overline{fb_S T^{\alpha}}(t) + \overline{fb_S A^{\alpha}}(t) + \overline{(1-f)b_S A}(t))}{\overline{b_S} - (1-\delta)\overline{fb_S Q^{\alpha}}(t) - \delta(\overline{fb_S I^{\alpha}}(t) + \overline{(1-f)b_S I}(t))}\\ & + \lambda b_S(1 - I_{a_S,b_S}(t) - P_{a_S,b_S}(t))\frac{\delta(\overline{fa_S P^{\alpha}}(t) + \overline{(1-f)a_S P}(t)) + (1-\delta)(\overline{fa_S T^{\alpha}}(t) + \overline{fa_S A^{\alpha}}(t) + \overline{(1-f)a_S A}(t))}{\overline{b_S} - (1-\delta)\overline{fb_S Q^{\alpha}}(t) - \delta(\overline{fb_S I^{\alpha}}(t) + \overline{(1-f)b_S I}(t))}\end{aligned} \tag{35}$$

$$\partial_t I^{\alpha}_{a_S,b_S}(t) = -\mu_I I^{\alpha}_{a_S,b_S}(t) + \gamma_P P^{\alpha}_{a_S,b_S}(t) \tag{36}$$

$$\begin{aligned}\partial_t P^{\alpha}_{a_S,b_S}(t) = & -\gamma_P P^{\alpha}_{a_S,b_S}(t)\\ & + \lambda a_S(1 - I^{\alpha}_{a_S,b_S}(t) - P^{\alpha}_{a_S,b_S}(t))\frac{\delta(\overline{fb_S P^{\alpha}}(t) + \overline{(1-f)b_S P}(t)) + (1-\delta)(\overline{fb_S T^{\alpha}}(t) + \overline{fb_S A^{\alpha}}(t) + \overline{(1-f)b_S A}(t))}{\overline{b_S} - (1-\delta)\overline{fb_S Q^{\alpha}}(t) - \delta(\overline{fb_S I^{\alpha}}(t) + \overline{(1-f)b_S I}(t))}\\ & + \lambda b_S(1 - I^{\alpha}_{a_S,b_S}(t) - P^{\alpha}_{a_S,b_S}(t))\frac{\delta(\overline{fa_S P^{\alpha}}(t) + \overline{(1-f)a_S P}(t)) + (1-\delta)(\overline{fa_S T^{\alpha}}(t) + \overline{fa_S A^{\alpha}}(t) + \overline{(1-f)a_S A}(t))}{\overline{b_S} - (1-\delta)\overline{fb_S Q^{\alpha}}(t) - \delta(\overline{fb_S I^{\alpha}}(t) + \overline{(1-f)b_S I}(t))}\end{aligned} \tag{37}$$

$$\begin{aligned}\partial_t A_{a_S,b_S}(t) = & -\mu A_{a_S,b_S}(t)\\ & + \lambda a_S(1 - A_{a_S,b_S}(t))\frac{\delta(\overline{fb_S P^{\alpha}}(t) + \overline{(1-f)b_S P}(t)) + (1-\delta)(\overline{fb_S T^{\alpha}}(t) + \overline{fb_S A^{\alpha}}(t) + \overline{(1-f)b_S A}(t))}{\overline{b_S} - (1-\delta)\overline{fb_S Q^{\alpha}}(t) - \delta(\overline{fb_S I^{\alpha}}(t) + \overline{(1-f)b_S I}(t))}\\ & + \lambda b_S(1 - A_{a_S,b_S}(t))\frac{\delta(\overline{fa_S P^{\alpha}}(t) + \overline{(1-f)a_S P}(t)) + (1-\delta)(\overline{fa_S T^{\alpha}}(t) + \overline{fa_S A^{\alpha}}(t) + \overline{(1-f)a_S A}(t))}{\overline{b_S} - (1-\delta)\overline{fb_S Q^{\alpha}}(t) - \delta(\overline{fb_S I^{\alpha}}(t) + \overline{(1-f)b_S I}(t))}\end{aligned} \tag{38}$$

$$\begin{aligned}\partial_t A^{\alpha}_{a_S,b_S}(t) = & -\mu A^{\alpha}_{a_S,b_S}(t)\\ & + \lambda a_S(1 - A^{\alpha}_{a_S,b_S}(t) - T^{\alpha}_{a_S,b_S}(t) - Q^{\alpha}_{a_S,b_S}(t))\frac{\delta\overline{(1-f)b_S P}(t) + (1-\delta)(\overline{fb_S T^{\alpha}}(t) + \overline{fb_S A^{\alpha}}(t) + \overline{(1-f)b_S A}(t))}{\overline{b_S} - (1-\delta)\overline{fb_S Q^{\alpha}}(t) - \delta(\overline{fb_S I^{\alpha}}(t) + \overline{(1-f)b_S I}(t))}\\ & + \lambda b_S(1 - A^{\alpha}_{a_S,b_S}(t) - T^{\alpha}_{a_S,b_S}(t) - Q^{\alpha}_{a_S,b_S}(t))\frac{\delta\overline{(1-f)a_S P}(t) + (1-\delta)(\overline{fa_S T^{\alpha}}(t) + \overline{fa_S A^{\alpha}}(t) + \overline{(1-f)a_S A}(t))}{\overline{b_S} - (1-\delta)\overline{fb_S Q^{\alpha}}(t) - \delta(\overline{fb_S I^{\alpha}}(t) + \overline{(1-f)b_S I}(t))}\\ & - \lambda\delta a_S A^{\alpha}_{a_S,b_S}(t)\frac{\overline{fb_S} - \overline{fb_S P^{\alpha}}(t) - \overline{fb_S I^{\alpha}}(t)}{\overline{b_S} - (1-\delta)\overline{fb_S Q^{\alpha}}(t) - \delta(\overline{fb_S I^{\alpha}}(t) + \overline{(1-f)b_S I}(t))}\\ & - \lambda\delta b_S A^{\alpha}_{a_S,b_S}(t)\frac{\overline{fa_S} - \overline{fa_S P^{\alpha}}(t) - \overline{fa_S I^{\alpha}}(t)}{\overline{b_S} - (1-\delta)\overline{fb_S Q^{\alpha}}(t) - \delta(\overline{fb_S I^{\alpha}}(t) + \overline{(1-f)b_S I}(t))}\end{aligned} \tag{39}$$

$$\partial_t Q^\alpha_{a_S,b_S}(t) = -\mu Q^\alpha_{a_S,b_S}(t) + \gamma_P T^\alpha_{a_S,b_S}(t) \tag{40}$$

$$\begin{aligned}\partial_t T^\alpha_{a_S,b_S}(t) =& -(\mu+\gamma_P)T^\alpha_{a_S,b_S}(t)\\ &+\lambda a_S(1-A^\alpha_{a_S,b_S}(t)-T^\alpha_{a_S,b_S}(t)-Q^\alpha_{a_S,b_S}(t))\frac{\delta\overline{fb_SP^\alpha}(t)}{\overline{b_S}-(1-\delta)\overline{fb_SQ^\alpha}(t)-\delta(\overline{fb_SI^\alpha}(t)+\overline{(1-f)b_SI}(t))}\\ &+\lambda b_S(1-A^\alpha_{a_S,b_S}(t)-T^\alpha_{a_S,b_S}(t)-Q^\alpha_{a_S,b_S}(t))\frac{\delta\overline{fa_SP^\alpha}(t)}{\overline{b_S}-(1-\delta)\overline{fb_SQ^\alpha}(t)-\delta(\overline{fb_SI^\alpha}(t)+\overline{(1-f)b_SI}(t))}\\ &+\lambda\delta a_S A^\alpha_{a_S,b_S}(t)\frac{\overline{fb_S}-\overline{fb_SP^\alpha}(t)-\overline{fb_SI^\alpha}(t)}{\overline{b_S}-(1-\delta)\overline{fb_SQ^\alpha}(t)-\delta(\overline{fb_SI^\alpha}(t)+\overline{(1-f)b_SI}(t))}\\ &+\lambda\delta b_S A^\alpha_{a_S,b_S}(t)\frac{\overline{fa_S}-\overline{fa_SP^\alpha}(t)-\overline{fa_SI^\alpha}(t)}{\overline{b_S}-(1-\delta)\overline{fb_SQ^\alpha}(t)-\delta(\overline{fb_SI^\alpha}(t)+\overline{(1-f)b_SI}(t))}\end{aligned} \tag{41}$$

This set of equations admits as a stationary state the absorbing state, a configuration where all the population is susceptible. To obtain the condition for the stability of the absorbing state, i.e. the epidemic threshold, we apply a linear stability analysis around the absorbing state.

Let us now consider the case of realistic correlations between the activity and attractiveness: $\rho(a_S, b_S) = \rho_S(a_S)\delta(b_S - a_S)$, with general $\rho_S(a_S)$. If we average on all activity classes, we obtain the temporal evolution of the average probabilities $\overline{P}(t)$, $\overline{I}(t)$, $\overline{A}(t)$, $\overline{P^\alpha}(t)$, $\overline{I^\alpha}(t)$, $\overline{A^\alpha}(t)$, $\overline{Q^\alpha}(t)$, $\overline{T^\alpha}(t)$; similarly we obtain the temporal evolution of $\overline{fa_SP^\alpha}(t)$, $\overline{fa_ST^\alpha}(t)$, $\overline{a_SA^\alpha}(t)$, $\overline{fa_SA^\alpha}(t)$, $\overline{(1-f)a_SP}(t)$, $\overline{(1-f)a_SA}(t)$, multiplying the equations for $f(a_S)a_S\rho_S(a_S)$ or $a_S\rho_S(a_S)$ or $(1-f(a_S))a_S\rho_S(a_S)$ and integrating over all activity classes. We neglect the terms of second order in probabilities obtaining a linearized set of 14 differential equations:

$$\partial_t\overline{I}(t) = -\mu_I\overline{I}(t) + \gamma_P\overline{P}(t) \tag{42}$$

$$\partial_t\overline{P}(t) = -\gamma_P\overline{P}(t) + 2\lambda[\delta(\overline{fa_SP^\alpha}(t)+\overline{(1-f)a_SP}(t)) + (1-\delta)(\overline{fa_ST^\alpha}(t)+\overline{fa_SA^\alpha}(t)+\overline{(1-f)a_SA}(t))] \tag{43}$$

$$\partial_t\overline{I^\alpha}(t) = -\mu_I\overline{I^\alpha}(t) + \gamma_P\overline{P^\alpha}(t) \tag{44}$$

$$\partial_t\overline{P^\alpha}(t) = -\gamma_P\overline{P^\alpha}(t) + 2\lambda[\delta(\overline{fa_SP^\alpha}(t)+\overline{(1-f)a_SP}(t)) + (1-\delta)(\overline{fa_ST^\alpha}(t)+\overline{fa_SA^\alpha}(t)+\overline{(1-f)a_SA}(t))] \tag{45}$$

$$\partial_t\overline{A}(t) = -\mu\overline{A}(t) + 2\lambda[\delta(\overline{fa_SP^\alpha}(t)+\overline{(1-f)a_SP}(t)) + (1-\delta)(\overline{fa_ST^\alpha}(t)+\overline{fa_SA^\alpha}(t)+\overline{(1-f)a_SA}(t))] \tag{46}$$

$$\partial_t\overline{Q^\alpha}(t) = -\mu\overline{Q^\alpha}(t) + \gamma_P\overline{T^\alpha}(t) \tag{47}$$

$$\partial_t\overline{T^\alpha}(t) = -(\mu+\gamma_P)\overline{T^\alpha}(t) + 2\lambda\delta\overline{fa_SP^\alpha}(t) + 2\lambda\delta\overline{a_SA^\alpha}(t)\frac{\overline{fa_S}}{\overline{a_S}} \tag{48}$$

$$\begin{aligned}\partial_t\overline{A^\alpha}(t) =& -\mu\overline{A^\alpha}(t) + 2\lambda[\delta\overline{(1-f)a_SP}(t) + (1-\delta)(\overline{fa_ST^\alpha}(t)+\overline{fa_SA^\alpha}(t)+\overline{(1-f)a_SA}(t))]\\ &-2\lambda\delta\overline{a_SA^\alpha}(t)\frac{\overline{fa_S}}{\overline{a_S}}\end{aligned} \tag{49}$$

$$\begin{aligned}\partial_t\overline{a_SA^\alpha}(t) =& -\mu\overline{a_SA^\alpha}(t) + 2\lambda\frac{\overline{a_S^2}}{\overline{a_S}}[\delta\overline{(1-f)a_SP}(t) + (1-\delta)(\overline{fa_ST^\alpha}(t)+\overline{fa_SA^\alpha}(t)+\overline{(1-f)a_SA}(t))]\\ &-2\lambda\delta\overline{a_S^2A^\alpha}(t)\frac{\overline{fa_S}}{\overline{a_S}}\end{aligned} \tag{50}$$

$$\begin{aligned}\partial_t\overline{fa_SP^\alpha}(t) =& -\gamma_P\overline{fa_SP^\alpha}(t)\\ &+2\lambda\frac{\overline{fa_S^2}}{\overline{a_S}}[\delta(\overline{fa_SP^\alpha}(t)+\overline{(1-f)a_SP}(t)) + (1-\delta)(\overline{fa_ST^\alpha}(t)+\overline{fa_SA^\alpha}(t)+\overline{(1-f)a_SA}(t))]\end{aligned} \tag{51}$$

$$\begin{aligned}\partial_t\overline{(1-f)a_SP}(t) =& -\gamma_P\overline{(1-f)a_SP}(t)\\ &+2\lambda\frac{\overline{(1-f)a_S^2}}{\overline{a_S}}[\delta(\overline{fa_SP^\alpha}(t)+\overline{(1-f)a_SP}(t)) + (1-\delta)(\overline{fa_ST^\alpha}(t)+\overline{fa_SA^\alpha}(t)+\overline{(1-f)a_SA}(t))]\end{aligned} \tag{52}$$

$$\begin{aligned}\partial_t\overline{(1-f)a_SA}(t) =& -\mu\overline{(1-f)a_SA}(t)\\ &+2\lambda\frac{\overline{(1-f)a_S^2}}{\overline{a_S}}[\delta(\overline{fa_SP^\alpha}(t)+\overline{(1-f)a_SP}(t)) + (1-\delta)(\overline{fa_ST^\alpha}(t)+\overline{fa_SA^\alpha}(t)+\overline{(1-f)a_SA}(t))]\end{aligned} \tag{53}$$

$$\partial_t\overline{fa_ST^\alpha}(t) = -(\mu+\gamma_P)\overline{fa_ST^\alpha}(t) + 2\lambda\delta\frac{\overline{fa_S^2}}{\overline{a_S}}\overline{fa_SP^\alpha}(t) + 2\lambda\delta\overline{fa_S^2A^\alpha}(t)\frac{\overline{fa_S}}{\overline{a_S}} \tag{54}$$

$$\begin{aligned}\partial_t\overline{fa_SA^\alpha}(t) =& -\mu\overline{fa_SA^\alpha}(t) + 2\lambda\frac{\overline{fa_S^2}}{\overline{a_S}}[\delta\overline{(1-f)a_SP}(t) + (1-\delta)(\overline{fa_ST^\alpha}(t)+\overline{fa_SA^\alpha}(t)+\overline{(1-f)a_SA}(t))]\\ &-2\lambda\delta\overline{fa_S^2A^\alpha}(t)\frac{\overline{fa_S}}{\overline{a_S}}\end{aligned} \tag{55}$$

Similarly to the manual CT, in order to close the equations and obtain a complete set of linearized equations, we express $\overline{fa_S^2A^\alpha}(t)$ and $\overline{a_S^2A^\alpha}(t)$ in terms of the other average probabilities using their own definition. By

definition $\overline{fa_S^2A^\alpha}(t) = \int da_S\rho_S(a_S)f(a_S)a_S^2A^\alpha_{a_S}(t)$ and $\overline{a_S^2A^\alpha}(t) = \int da_S\rho_S(a_S)a_S^2A^\alpha_{a_S}(t)$: since we are interested in studying the steady state and its stability, we consider the Eq. (39) for $\rho(a_S, b_S) = \rho_S(a_S)\delta(b_S - a_S)$, linearized around the absorbing state and near to the stationary condition $\partial_t A^\alpha_{a_S}(t) \sim 0$:

$$A^\alpha_{a_S}(t) \simeq \frac{2\lambda a_S[\delta\overline{(1-f)a_SP}(t) + (1-\delta)(\overline{fa_ST^\alpha}(t) + \overline{fa_SA^\alpha}(t) + \overline{(1-f)a_SA}(t))]}{\mu\overline{a_S} + 2\lambda\delta a_S\overline{fa_S}} \tag{56}$$

Thus,

$$\overline{fa_S^2A^\alpha}(t) \simeq \frac{2}{\overline{a_S}}r[\delta\overline{(1-f)a_SP}(t) + (1-\delta)(\overline{fa_ST^\alpha}(t) + \overline{fa_SA^\alpha}(t) + \overline{(1-f)a_SA}(t))]H \tag{57}$$

$$\overline{a_S^2A^\alpha}(t) \simeq \frac{2}{\overline{a_S}}r[\delta\overline{(1-f)a_SP}(t) + (1-\delta)(\overline{fa_ST^\alpha}(t) + \overline{fa_SA^\alpha}(t) + \overline{(1-f)a_SA}(t))]Z \tag{58}$$

where $H = \overline{\frac{fa_S^3}{1+2r\delta a_S\frac{\overline{fa_S}}{\overline{a_S}}}} = \int da_S\rho_S(a_S)\frac{f(a_S)a_S^3}{1+2r\delta a_S\frac{\overline{fa_S}}{\overline{a_S}}}$, $Z = \overline{\frac{a_S^3}{1+2r\delta a_S\frac{\overline{fa_S}}{\overline{a_S}}}}$.

In this way we obtain the following linearized equations for $\overline{fa_ST^\alpha}$, $\overline{fa_SA^\alpha}$ and $\overline{a_SA^\alpha}$ near the absorbing stationary state:

$$\begin{aligned}\partial_t\overline{a_SA^\alpha}(t) = & -\mu\overline{a_SA^\alpha}(t) + 2\lambda\frac{\overline{a_S^2}}{\overline{a_S}}[\delta\overline{(1-f)a_SP}(t) + (1-\delta)(\overline{fa_ST^\alpha}(t) + \overline{fa_SA^\alpha}(t) + \overline{(1-f)a_SA}(t))] \\ & - 4\lambda r\delta\frac{\overline{fa_S}}{\overline{a_S}^2}[\delta\overline{(1-f)a_SP}(t) + (1-\delta)(\overline{fa_ST^\alpha}(t) + \overline{fa_SA^\alpha}(t) + \overline{(1-f)a_SA}(t))]Z\end{aligned} \tag{59}$$

$$\begin{aligned}\partial_t\overline{fa_ST^\alpha}(t) = & -(\mu+\gamma_P)\overline{fa_ST^\alpha}(t) + 2\lambda\delta\frac{\overline{fa_S^2}}{\overline{a_S}}\overline{fa_SP^\alpha}(t) \\ & + 4\lambda r\delta\frac{\overline{fa_S}}{\overline{a_S}^2}[\delta\overline{(1-f)a_SP}(t) + (1-\delta)(\overline{fa_ST^\alpha}(t) + \overline{fa_SA^\alpha}(t) + \overline{(1-f)a_SA}(t))]H\end{aligned} \tag{60}$$

$$\begin{aligned}\partial_t\overline{fa_SA^\alpha}(t) = & -\mu\overline{fa_SA^\alpha}(t) + 2\lambda\frac{\overline{fa_S^2}}{\overline{a_S}}[\delta\overline{(1-f)a_SP}(t) + (1-\delta)(\overline{fa_ST^\alpha}(t) + \overline{fa_SA^\alpha}(t) + \overline{(1-f)a_SA}(t))] \\ & - 4\lambda r\delta\frac{\overline{fa_S}}{\overline{a_S}^2}[\delta\overline{(1-f)a_SP}(t) + (1-\delta)(\overline{fa_ST^\alpha}(t) + \overline{fa_SA^\alpha}(t) + \overline{(1-f)a_SA}(t))]H\end{aligned} \tag{61}$$

Focusing on the Jacobian matrix of this set of 14 linearized equations:

$$J = \begin{bmatrix} -\mu_I & \gamma_P & 0 & 0 & 0 & 0 & 0 & 0 & 0 & 0 & 0 & 0 & 0 & 0 \\ 0 & -\gamma_P & 0 & 0 & 0 & 0 & 0 & 0 & 0 & 2\lambda\delta & 2\lambda\delta & 2\lambda(1-\delta) & 2\lambda(1-\delta) & 2\lambda(1-\delta) \\ 0 & 0 & -\mu_I & \gamma_P & 0 & 0 & 0 & 0 & 0 & 0 & 0 & 0 & 0 & 0 \\ 0 & 0 & 0 & -\gamma_P & 0 & 0 & 0 & 0 & 0 & 2\lambda\delta & 2\lambda\delta & 2\lambda(1-\delta) & 2\lambda(1-\delta) & 2\lambda(1-\delta) \\ 0 & 0 & 0 & 0 & -\mu & 0 & 0 & 0 & 0 & 2\lambda\delta & 2\lambda\delta & 2\lambda(1-\delta) & 2\lambda(1-\delta) & 2\lambda(1-\delta) \\ 0 & 0 & 0 & 0 & 0 & -\mu & +\gamma_P & 0 & 0 & 0 & 0 & 0 & 0 & 0 \\ 0 & 0 & 0 & 0 & 0 & 0 & -\mu-\gamma_P & 0 & 2\lambda\delta\frac{\overline{fa_S}}{\overline{a_S}} & 2\lambda\delta & 0 & 0 & 0 & 0 \\ 0 & 0 & 0 & 0 & 0 & 0 & 0 & -\mu & -2\lambda\delta\frac{\overline{fa_S}}{\overline{a_S}} & 0 & 2\lambda\delta & 2\lambda(1-\delta) & 2\lambda(1-\delta) & 2\lambda(1-\delta) \\ 0 & 0 & 0 & 0 & 0 & 0 & 0 & 0 & -\mu & 0 & \Delta+\phi-\Phi\frac{Z}{H} & \theta+\Gamma-\Psi\frac{Z}{H} & \theta+\Gamma-\Psi\frac{Z}{H} & \theta+\Gamma-\Psi\frac{Z}{H} \\ 0 & 0 & 0 & 0 & 0 & 0 & 0 & 0 & 0 & -\gamma_P+\Delta & \Delta & \Gamma & \Gamma & \Gamma \\ 0 & 0 & 0 & 0 & 0 & 0 & 0 & 0 & 0 & \phi & -\gamma_P+\phi & \theta & \theta & \theta \\ 0 & 0 & 0 & 0 & 0 & 0 & 0 & 0 & 0 & \phi & \phi & -\mu+\theta & \theta & \theta \\ 0 & 0 & 0 & 0 & 0 & 0 & 0 & 0 & 0 & \Delta & \Phi & \Psi & -\mu-\gamma_P+\Psi & \Psi \\ 0 & 0 & 0 & 0 & 0 & 0 & 0 & 0 & 0 & 0 & \Delta-\Phi & \Gamma-\Psi & \Gamma-\Psi & -\mu+\Gamma-\Psi \end{bmatrix}$$
$$= \begin{bmatrix} \mathbb{A}(9\text{x}9) & \mathbb{C}(9\text{x}5) \\ \mathbb{O}(5\text{x}9) & \mathbb{B}(5\text{x}5) \end{bmatrix} \tag{62}$$

where $\Delta = 2\lambda\delta\frac{\overline{fa_S^2}}{\overline{a_S}}$, $\Gamma = 2\lambda(1-\delta)\frac{\overline{fa_S^2}}{\overline{a_S}}$, $\phi = 2\lambda\delta\frac{\overline{(1-f)a_S^2}}{\overline{a_S}}$, $\theta = 2\lambda(1-\delta)\frac{\overline{(1-f)a_S^2}}{\overline{a_S}}$, $\Phi = 4\lambda r\delta^2\frac{\overline{fa_S}}{\overline{a_S}^2}H$ and $\Psi = 4\lambda r\delta(1-\delta)\frac{\overline{fa_S}}{\overline{a_S}^2}H$.

The Jacobian matrix is a block matrix and we can consider separately the two blocks on the diagonal: for the first block $\mathbb{A}$ it is evident that the eigenvalues are $\xi_{1,2} = -\mu_I$, $\xi_{3,4,5,6} = -\mu$, $\xi_{7,8} = -\gamma_P$, $\xi_9 = -\mu-\gamma_P$, all negative. Therefore, it is sufficient to study block $\mathbb{B}$, which is a matrix 5x5. The characteristic polynomial of

$\mathbb{B}$ is a polynomial of degree 5: we apply the Descartes' rule of signs, to impose all roots to be negative and we obtain the condition for the stability of the absorbing state:

$$8r^3\delta^2(1-\delta)\frac{\overline{fa_S^2}\,\overline{fa_S}}{\overline{a_S}}\overline{\frac{fa_S^3}{1+2r\delta a_S\frac{\overline{fa_S}}{\overline{a_S}}}}\frac{\gamma_P}{\mu}-4r^2\delta(1-\delta)\left[\overline{fa_S^2}^2+\frac{\gamma_P}{\mu}\overline{fa_S}\,\overline{\frac{fa_S^3}{1+2r\delta a_S\frac{\overline{fa_S}}{\overline{a_S}}}}\right]\frac{\gamma_P}{\mu} + 2r\overline{a_S^2}\,\overline{a_S}\left(\frac{\gamma_P}{\mu}+1\right)\left(\delta+\frac{\gamma_P}{\mu}(1-\delta)\right)-\overline{a_S}^2\frac{\gamma_P}{\mu}\left(\frac{\gamma_P}{\mu}+1\right)<0 \tag{63}$$

By setting the equality, the equation allows to obtain a closed relation for an estimate of the epidemic threshold. The epidemic threshold obtained with the mean-field approach is exact and it holds for digital contact tracing, with arbitrary $\rho_S(a_S)$ and $f(a_S)$.

C. Hybrid CT

We now focus on the hybrid CT, with simultaneous implementation of both digital and manual CT. The transitions occurring in this case can be deduced starting from the transitions described above for manual and digital CT. A relevant difference is the need to consider an additional compartment, T^α_M, indicating individual endowed with the app that are nevertheless traced manually, so that their transition to the Q^α state occurs with a rate γ_A. In a manner analogous to the previous cases, we apply the *activity-attractiveness based mean-field* approach to the hybrid CT. At the mean-field level, the epidemic dynamics is described by the probabilities defined for the digital CT, with these additional/redefined probabilities:

- $T^\alpha_{a_S,b_S|M}(t)$ for an asymptomatic node with app to be infected and traced manually;
- $T_{a_S,b_S}(t)$ for an asymptomatic node without the app to be infected and traced manually;
- $Q_{a_S,b_S}(t)$ for an asymptomatic node without the app to be isolated;
- $1-A_{a_S,b_S}(t)-Q_{a_S,b_S}(t)-T_{a_S,b_S}(t)$ to be susceptible at time t, for a node without app and which will not develop symptoms;
- $1-A^\alpha_{a_S,b_S}(t)-Q^\alpha_{a_S,b_S}(t)-T^\alpha_{a_S,b_S}(t)-T^\alpha_{a_S,b_S|M}(t)$ to be susceptible at time t, for a node with app and which will not develop symptoms;

We consider the system in the thermodynamic limit and for $\rho(a_S,b_S)=\rho_S(a_S)\delta(b_S-a_S)$: in this case the average attractiveness at time t is $\langle b(t)\rangle=\overline{a_S}-(1-\delta)(\overline{fa_SQ^\alpha}(t)+\overline{(1-f)a_SQ}(t))-\delta(\overline{fa_SI^\alpha}(t)+\overline{(1-f)a_SI}(t))$. The probabilities introduced evolve according to the following equations, obtained analogously to those for manual and digital CT:

$$\partial_t P_{a_S}(t)=-\gamma_P P_{a_S}(t)+2\lambda(1-P_{a_S}(t)-I_{a_S}(t))\frac{a_S}{\langle b(t)\rangle}[\delta(\overline{a_SfP^\alpha}(t)+\overline{a_S(1-f)P}(t)) + (1-\delta)(\overline{a_SfA^\alpha}(t)+\overline{a_SfT^\alpha}(t)+\overline{a_SfT^\alpha_M}(t)+\overline{a_S(1-f)T}(t)+\overline{a_S(1-f)A}(t))] \tag{64}$$

$$\partial_t I_{a_S}(t)=-\mu_I I_{a_S}(t)+\gamma_P P_{a_S}(t) \tag{65}$$

$$\partial_t P^\alpha_{a_S}(t)=-\gamma_P P^\alpha_{a_S}(t)+2\lambda(1-P^\alpha_{a_S}(t)-I^\alpha_{a_S}(t))\frac{a_S}{\langle b(t)\rangle}[\delta(\overline{a_SfP^\alpha}(t)+\overline{a_S(1-f)P}(t)) + (1-\delta)(\overline{a_SfA^\alpha}(t)+\overline{a_SfT^\alpha}(t)+\overline{a_SfT^\alpha_M}(t)+\overline{a_S(1-f)T}(t)+\overline{a_S(1-f)A}(t))] \tag{66}$$

$$\partial_t I^\alpha_{a_S}(t)=-\mu_I I^\alpha_{a_S}(t)+\gamma_P P^\alpha_{a_S}(t) \tag{67}$$

$$\partial_t A_{a_S}(t)=-\mu A_{a_S}(t)+2\lambda(1-A_{a_S}(t)-T_{a_S}(t)-Q_{a_S}(t))\frac{a_S}{\langle b(t)\rangle}[\delta(\overline{a_S(1-\varepsilon)fP^\alpha}(t)+\overline{a_S(1-\varepsilon)(1-f)P}(t)) + (1-\delta)(\overline{a_SfA^\alpha}(t)+\overline{a_SfT^\alpha}(t)+\overline{a_SfT^\alpha_M}(t)+\overline{a_S(1-f)T}(t)+\overline{a_S(1-f)A}(t))] - 2\lambda\delta A_{a_S}(t)\frac{a_S}{\langle b(t)\rangle}[(\overline{a_S\varepsilon f}-\overline{a_S\varepsilon fP^\alpha}(t)-\overline{a_S\varepsilon fI^\alpha}(t))+(\overline{a_S\varepsilon(1-f)}-\overline{a_S\varepsilon(1-f)P}(t)-\overline{a_S\varepsilon(1-f)I}(t))] \tag{68}$$

$$\partial_t T_{a_S}(t)=-(\mu+\gamma_A)T_{a_S}(t)+2\lambda\delta\frac{a_S}{\langle b(t)\rangle}(1-A_{a_S}(t)-T_{a_S}(t)-Q_{a_S}(t))[\overline{a_S\varepsilon fP^\alpha}(t)+\overline{a_S\varepsilon(1-f)P}(t)] + 2\lambda\delta A_{a_S}(t)\frac{a_S}{\langle b(t)\rangle}[(\overline{a_S\varepsilon f}-\overline{a_S\varepsilon fP^\alpha}(t)-\overline{a_S\varepsilon fI^\alpha}(t))+(\overline{a_S\varepsilon(1-f)}-\overline{a_S\varepsilon(1-f)P}(t)-\overline{a_S\varepsilon(1-f)I}(t))] \tag{69}$$

$$\partial_t Q_{a_S}(t)=-\mu Q_{a_S}(t)+\gamma_A T_{a_S}(t) \tag{70}$$

$$
\begin{aligned}
\partial_t A^\alpha_{a_S}(t) = & -\mu A^\alpha_{a_S}(t) + 2\lambda(1 - A^\alpha_{a_S}(t) - T^\alpha_{a_S}(t) - T^\alpha_{a_S|M}(t) - Q^\alpha_{a_S}(t))\frac{a_S}{\langle b(t)\rangle}[\delta\overline{a_S(1-\varepsilon)(1-f)P}(t) \\
& + (1-\delta)(\overline{a_S f A^\alpha}(t) + \overline{a_S f T^\alpha}(t) + \overline{a_S f T^\alpha_M}(t) + \overline{a_S(1-f)T}(t) + \overline{a_S(1-f)A}(t))] \\
& - 2\lambda\delta A^\alpha_{a_S}(t)\frac{a_S}{\langle b(t)\rangle}[(\overline{a_S f} - \overline{a_S f P^\alpha}(t) - \overline{a_S f I^\alpha}(t)) + (\overline{a_S\varepsilon(1-f)} - \overline{a_S\varepsilon(1-f)P}(t) - \overline{a_S\varepsilon(1-f)I}(t))]
\end{aligned} \tag{71}
$$

$$
\begin{aligned}
\partial_t T^\alpha_{a_S}(t) = & -(\mu+\gamma_P)T^\alpha_{a_S}(t) + 2\lambda\delta\frac{a_S}{\langle b(t)\rangle}(1 - A^\alpha_{a_S}(t) - T^\alpha_{a_S}(t) - T^\alpha_{a_S|M}(t) - Q^\alpha_{a_S}(t))\overline{a_S f P^\alpha}(t) \\
& + 2\lambda\delta A^\alpha_{a_S}(t)\frac{a_S}{\langle b(t)\rangle}[\overline{a_S f} - \overline{a_S f P^\alpha}(t) - \overline{a_S f I^\alpha}(t)]
\end{aligned} \tag{72}
$$

$$
\begin{aligned}
\partial_t T^\alpha_{a_S|M}(t) = & -(\mu+\gamma_A)T^\alpha_{a_S|M}(t) + 2\lambda\delta\frac{a_S}{\langle b(t)\rangle}(1 - A^\alpha_{a_S}(t) - T^\alpha_{a_S}(t) - T^\alpha_{a_S|M}(t) - Q^\alpha_{a_S}(t))\overline{a_S\varepsilon(1-f)P}(t) \\
& + 2\lambda\delta A^\alpha_{a_S}(t)\frac{a_S}{\langle b(t)\rangle}[\overline{a_S\varepsilon(1-f)} - \overline{a_S\varepsilon(1-f)P}(t) - \overline{a_S\varepsilon(1-f)I}(t)]
\end{aligned} \tag{73}
$$

$$
\partial_t Q^\alpha_{a_S}(t) = -\mu Q^\alpha_{a_S}(t) + \gamma_P T^\alpha_{a_S}(t) + \gamma_A T^\alpha_{a_S|M}(t) \tag{74}
$$

This set of equations admits as a stationary state the absorbing state: to obtain the epidemic threshold, we apply a linear stability analysis around the absorbing state. We consider the temporal evolution of the probabilities averaged on the activity (eventually multiplied by combinations of a_S, $\varepsilon(a_S)$ and $f(a_S)$), obtaining a closed and complete set of 22 non-linear differential equations. The set of equations can be linearized obtaining the following Jacobian matrix:

$$
J = \begin{bmatrix} \mathbb{A}(13\text{x}13) & \mathbb{C}(13\text{x}9) \\ \mathbb{O}(9\text{x}13) & \mathbb{B}(9\text{x}9) \end{bmatrix} \tag{75}
$$

where $\mathbb{O}$ is the zero matrix.

$$
\mathbb{A} = \begin{bmatrix}
-\mu_I & \gamma_P & 0 & 0 & 0 & 0 & 0 & 0 & 0 & 0 & 0 & 0 & 0 \\
0 & -\gamma_P & 0 & 0 & 0 & 0 & 0 & 0 & 0 & 0 & 0 & 0 & 0 \\
0 & 0 & -\mu_I & \gamma_P & 0 & 0 & 0 & 0 & 0 & 0 & 0 & 0 & 0 \\
0 & 0 & 0 & -\gamma_P & 0 & 0 & 0 & 0 & 0 & 0 & 0 & 0 & 0 \\
0 & 0 & 0 & 0 & -\mu & \gamma_A & 0 & 0 & 0 & 0 & 0 & 0 & 0 \\
0 & 0 & 0 & 0 & 0 & -\mu-\gamma_A & 0 & 0 & 0 & 0 & 0 & 2\lambda\delta\frac{\overline{a_S\varepsilon}}{\overline{a_S}} & 0 \\
0 & 0 & 0 & 0 & 0 & 0 & -\mu & 0 & 0 & 0 & 0 & -2\lambda\delta\frac{\overline{a_S\varepsilon}}{\overline{a_S}} & 0 \\
0 & 0 & 0 & 0 & 0 & 0 & 0 & -\mu & \gamma_P & \gamma_A & 0 & 0 & 0 \\
0 & 0 & 0 & 0 & 0 & 0 & 0 & 0 & -\mu-\gamma_P & 0 & 0 & 0 & 2\lambda\delta\frac{\overline{a_S f}}{\overline{a_S}} \\
0 & 0 & 0 & 0 & 0 & 0 & 0 & 0 & 0 & -\mu-\gamma_A & 0 & 0 & 2\lambda\delta\frac{\overline{a_S\varepsilon(1-f)}}{\overline{a_S}} \\
0 & 0 & 0 & 0 & 0 & 0 & 0 & 0 & 0 & 0 & -\mu & 0 & -2\lambda\delta\frac{\overline{a_S f + a_S\varepsilon(1-f)}}{\overline{a_S}} \\
0 & 0 & 0 & 0 & 0 & 0 & 0 & 0 & 0 & 0 & 0 & -\mu & 0 \\
0 & 0 & 0 & 0 & 0 & 0 & 0 & 0 & 0 & 0 & 0 & 0 & -\mu
\end{bmatrix} \tag{76}
$$

$$
\mathbb{C} = \begin{bmatrix}
0 & 0 & 0 & 0 & 0 & 0 & 0 & 0 & 0 \\
0 & 0 & 2\lambda\delta & 2\lambda\delta & 2\lambda(1-\delta) & 2\lambda(1-\delta) & 2\lambda(1-\delta) & 2\lambda(1-\delta) & 2\lambda(1-\delta) \\
0 & 0 & 0 & 0 & 0 & 0 & 0 & 0 & 0 \\
0 & 0 & 2\lambda\delta & 2\lambda\delta & 2\lambda(1-\delta) & 2\lambda(1-\delta) & 2\lambda(1-\delta) & 2\lambda(1-\delta) & 2\lambda(1-\delta) \\
0 & 0 & 0 & 0 & 0 & 0 & 0 & 0 & 0 \\
2\lambda\delta & 2\lambda\delta & 0 & 0 & 0 & 0 & 0 & 0 & 0 \\
-2\lambda\delta & -2\lambda\delta & 2\lambda\delta & 2\lambda\delta & 2\lambda(1-\delta) & 2\lambda(1-\delta) & 2\lambda(1-\delta) & 2\lambda(1-\delta) & 2\lambda(1-\delta) \\
0 & 0 & 0 & 0 & 0 & 0 & 0 & 0 & 0 \\
0 & 0 & 0 & 2\lambda\delta & 0 & 0 & 0 & 0 & 0 \\
2\lambda\delta & 0 & 0 & 0 & 0 & 0 & 0 & 0 & 0 \\
-2\lambda\delta & 0 & 2\lambda\delta & 0 & 2\lambda(1-\delta) & 2\lambda(1-\delta) & 2\lambda(1-\delta) & 2\lambda(1-\delta) & 2\lambda(1-\delta) \\
-\Psi\delta & -\Psi\delta & \Psi\delta & \Psi\delta & \Psi(1-\delta) & \Psi(1-\delta) & \Psi(1-\delta) & \Psi(1-\delta) & \Psi(1-\delta) \\
-\theta\delta & 0 & \theta\delta & 0 & \theta(1-\delta) & \theta(1-\delta) & \theta(1-\delta) & \theta(1-\delta) & \theta(1-\delta)
\end{bmatrix} \tag{77}
$$

with $\Psi = 2\lambda\frac{\overline{a_S^2}}{\overline{a_S}} - 4\lambda r\delta\frac{\overline{a_S\varepsilon}}{\overline{a_S}^2}K$, $\theta = 2\lambda\frac{\overline{a_S^2}}{\overline{a_S}} - 4\lambda r\delta\frac{\overline{a_S\varepsilon(1-f)}+\overline{a_S f}}{\overline{a_S}^2}J$, $K = \overline{\frac{a_S^3}{1+2ra_S\delta\frac{\overline{a_S\varepsilon}}{\overline{a_S}}}}$ and $J = \overline{\frac{a_S^3}{1+2ra_S\delta\frac{\overline{a_S f}+\overline{a_S\varepsilon(1-f)}}{\overline{a_S}}}}$.

$$\mathbb{B} = \begin{bmatrix} -\gamma_P & 0 & \Xi\delta & \Xi\delta & \Xi\sigma & \Xi\sigma & \Xi\sigma & \Xi\sigma & \Xi\sigma \\ 0 & -\gamma_P & \Pi\delta & \Pi\delta & \Pi\sigma & \Pi\sigma & \Pi\sigma & \Pi\sigma & \Pi\sigma \\ 0 & 0 & -\gamma_P+\Delta\delta & \Delta\delta & \Delta\sigma & \Delta\sigma & \Delta\sigma & \Delta\sigma & \Delta\sigma \\ 0 & 0 & \beta\delta & -\gamma_P+\beta\delta & \beta\sigma & \beta\sigma & \beta\sigma & \beta\sigma & \beta\sigma \\ \Omega\delta & \Omega\delta & \phi\delta & \phi\delta & -\mu-\gamma_A+\phi\sigma & \phi\sigma & \phi\sigma & \phi\sigma & \phi\sigma \\ -\Gamma\delta & 0 & \Gamma\delta & \beta\delta & \Gamma\sigma & -\mu-\gamma_P+\Gamma\sigma & \Gamma\sigma & \Gamma\sigma & \Gamma\sigma \\ \Sigma\delta & 0 & \Phi\delta & 0 & \Phi\sigma & \Phi\sigma & -\mu-\gamma_A+\Phi\sigma & \Phi\sigma & \Phi\sigma \\ -\Omega\delta & -\Omega\delta & \Omega\delta & \Omega\delta & \Omega\sigma & \Omega\sigma & \Omega\sigma & -\mu+\Omega\sigma & \Omega\sigma \\ -\Lambda\delta & 0 & \Lambda\delta & 0 & \Lambda\sigma & \Lambda\sigma & \Lambda\sigma & \Lambda\sigma & -\mu+\Lambda\sigma \end{bmatrix} \tag{78}$$

with $\sigma = (1-\delta)$, $\Xi = 2\lambda\frac{\overline{a_S^2\varepsilon(1-f)}}{\overline{a_S}}$, $\Pi = 2\lambda\frac{\overline{a_S^2\varepsilon f}}{\overline{a_S}}$, $\Delta = 2\lambda\frac{\overline{a_S^2(1-f)}}{\overline{a_S}}$, $\beta = 2\lambda\frac{\overline{a_S^2 f}}{\overline{a_S}}$, $\phi = 4\lambda r\delta\frac{\overline{a_S\varepsilon}}{\overline{a_S}^2}H$, $\Gamma = 4\lambda r\delta\frac{\overline{a_S f}}{\overline{a_S}^2}Y$, $\Phi = 4\lambda r\delta\frac{\overline{a_S\varepsilon(1-f)}}{\overline{a_S}^2}Y$, $H = \overline{\frac{(1-f)a_S^3}{1+2r\delta a_S\frac{\overline{a_S\varepsilon}}{\overline{a_S}}}}$, $Y = \overline{\frac{f a_S^3}{1+2r\delta a_S\frac{\overline{a_S\varepsilon(1-f)}+\overline{a_S f}}{\overline{a_S}}}}$, $\Omega = \Delta - \phi$, $\Sigma = \beta - \Phi$ and $\Lambda = -\Gamma - \Phi + \beta$.

The Jacobian matrix is a block matrix and we can consider separately the two blocks on the diagonal: the first block $\mathbb{A}$ is triangular and the eigenvalues are $\xi_{1,2} = -\mu_I$, $\xi_{3,4,5,6,7,8} = -\mu$, $\xi_{9,10} = -\gamma_P$, $\xi_{11} = -\mu - \gamma_P$, $\xi_{12,13} = -\mu - \gamma_A$ all negative. Therefore, it is sufficient to study block $\mathbb{B}$, which is a matrix 9x9. The epidemic threshold in the hybrid case is therefore obtained by numerically diagonalizing the matrix $\mathbb{B}$ and imposing all its eigenvalues to be negative: this allows to obtain analytically the epidemic threshold for the hybrid CT for arbitrary $\rho_S(a_S)$, $\varepsilon(a_S)$ and $f(a_S)$.

D. Limit cases

The obtained closed relations for the stability of the absorbing state in the manual and digital CT hold for arbitrary $\rho_S(a_S)$, for completely general $f(a_S)$ and $\varepsilon(a_S)$ and for general delays: this allows to introduce complicated effects, such as delay in isolation, activity heterogeneities and limited scalability of the system.

Due to the complicated structure of the equations for the stability (Eq. (25) and Eq. (63)), it is possible to derive the epidemic threshold r_C only by solving the equation numerically. However, there are some simple limit cases in which the equations are considerably simplified, allowing to obtain the epidemic threshold in an explicit analytic form.

1. *Non-adaptive case (NA)*

Here we consider the non-adaptive case, in which no adaptive behaviour are implemented, i.e. infected nodes behave as if they were susceptible, with $(a_I, b_I) = (a_S, b_S)$. Thus, in this case $f(a_S) = \varepsilon(a_S) = 0$, $\forall a_S$ and $\gamma_P/\mu = 1$. Replacing these values either in Eq. (25) or in Eq. (63) we obtain, as expected:

$$2r\overline{a_S^2}\,\overline{a_S} - \overline{a_S}^2 = 0$$

So we obtain an explicit form for the epidemic threshold r_C in the non-adaptive case:

$$r_C^{NA} = \frac{\overline{a_S}}{2\overline{a_S^2}} \tag{79}$$

which is the Eq. (3) in the main paper. It reproduces the results previously obtained in Refs. [3, 5].

2. *Isolation of only symptomatic nodes*

Here we consider the case in which only symptomatic nodes are isolated as soon as they develops symptoms, i.e. no CT is implemented. Thus, in this case $f(a_S) = \varepsilon(a_S) = 0$, $\forall a_S$, while $(a_I, b_I) = (0, 0)$. Replacing these values either in Eq. (25) or in Eq. (63) we obtain, as expected:

$$2r\overline{a_S^2}\,\overline{a_S}\left(\delta + (1-\delta)\frac{\gamma_P}{\mu}\right) - \overline{a_S}^2\frac{\gamma_P}{\mu} = 0$$

So we obtain an explicit form for the epidemic threshold r_C:

$$r_C^{SYMPTO} = r_C^{NA} \frac{\frac{\gamma_P}{\mu}}{\delta + (1-\delta)\frac{\gamma_P}{\mu}} \tag{80}$$

which is the Eq. (4) in the main paper. For instantaneous symptoms development $\gamma_P/\mu \to \infty$ it reproduces the results previously obtained in Ref. [5], and for $\gamma_P/\mu = 1$ it reproduces the NA case (Eq. (79)).

3. *Homogeneous case without delays and without limited scalability*

Here we consider the case in which the population is homogeneous, i.e. $\rho(a_S, b_S) = \delta(a_S - a)\delta(b_S - b)$, with constant probability of downloading the app in digital CT, i.e. $f(a_S) = f$, and constant probability for a contact to be traced in manual CT, i.e. $\varepsilon(a_S) = \varepsilon$. Moreover we assume $\tau_C = 0$, that is $\gamma_A = \gamma_P$. Replacing these values in the Eq. (25), for the manual CT we obtain a quadratic equation in r:

$$4a^2\delta^2\varepsilon r^2 + 2a\left(\delta + \frac{\gamma_P}{\mu}(1-\delta-\delta\varepsilon)\right) r - \frac{\gamma_P}{\mu} = 0$$

The equation can be solved and we obtain:

$$r_C^{MANUAL} = r_C^{NA} \frac{2\frac{\gamma_P}{\mu}}{\delta + (1-\delta-\varepsilon\delta)\frac{\gamma_P}{\mu} + \sqrt{(\delta + (1-\delta-\varepsilon\delta)\frac{\gamma_P}{\mu})^2 + 4\delta^2\varepsilon\frac{\gamma_P}{\mu}}} \tag{81}$$

which is the Eq. (5) in the main paper.

Analogously, replacing the values in the Eq. (63) for the digital CT we obtain an equation of second degree in r:

$$4a^2\delta f\left(\delta + \frac{\gamma_P}{\mu}(1-\delta)(1-f)\right) r^2 + 2a\left(\delta + \frac{\gamma_P}{\mu}(1-\delta-\delta f)\right) r - \frac{\gamma_P}{\mu} = 0$$

The equation can be solved and we obtain:

$$r_C^{APP} = r_C^{NA} \frac{2\frac{\gamma_P}{\mu}}{\delta + (1-\delta-f\delta)\frac{\gamma_P}{\mu} + \sqrt{(\delta + (1-\delta-f\delta)\frac{\gamma_P}{\mu})^2 + 4\delta f\frac{\gamma_P}{\mu}(\delta + \frac{\gamma_P}{\mu}(1-f)(1-\delta))}} \tag{82}$$

which is the Eq. (6) in the main paper.

4. *Heterogeneous case*

Hereafter and in the main text we consider a realistic heterogeneous population, i.e. a power-law activity distribution:

$$\rho(a_S, b_S) \sim a_S^{-(\nu+1)}\delta(b_S - a_S) \tag{83}$$

with $a_S \in [a_m, a_M]$ and $\eta = a_M/a_m$, where a_m and a_M are respectively activity lower and upper cut-off.

Supplementary Fig. 2 shows that the considered distribution is maximally broad for $\nu = 1$: indeed, the ratio $\overline{a_S^2}/\overline{a_S}^2$, which we plot, provides a estimate of the heterogeneity and fluctuations of the distribution and it is maximized for $\nu = 1$. On the contrary for $\nu \to 0$ the distribution is counterintuitively more homogeneous, due to cut-off effects and constraints imposed on the distribution ($\overline{a_S}$ and η fixed), which thus set the maximum heterogeneous condition at $\nu = 1$.

In this heterogeneous case it is possible to derive the epidemic threshold r_C only numerically, due to the complex structure of the equations for the stability (Eq. (25) and Eq. (63)). Fig. 3(a,b) and Fig. 4(a,b) of the main text show that both manual and digital CT protocols are more effective when the population is heterogeneous, indeed the maximum gain in the epidemic threshold is for $\nu \sim 1 - 1.5$. However, the maximum does not occur exactly at $\nu = 1$, i.e. when the distribution is maximally broad. Indeed, the epidemic thresholds do not depend solely on activity fluctuations, i.e. $\overline{a_S^2}$, but also on higher order moments (as $\overline{a_S^3}$), as shown by Eq. (25) and Eq. (63). Thus, the CT protocols are more effective in heterogeneous populations and the maximum gain in r_C also depends on higher order moments of ρ_S and on other model parameters, as shown in Supplementary Fig. 3(b-c).

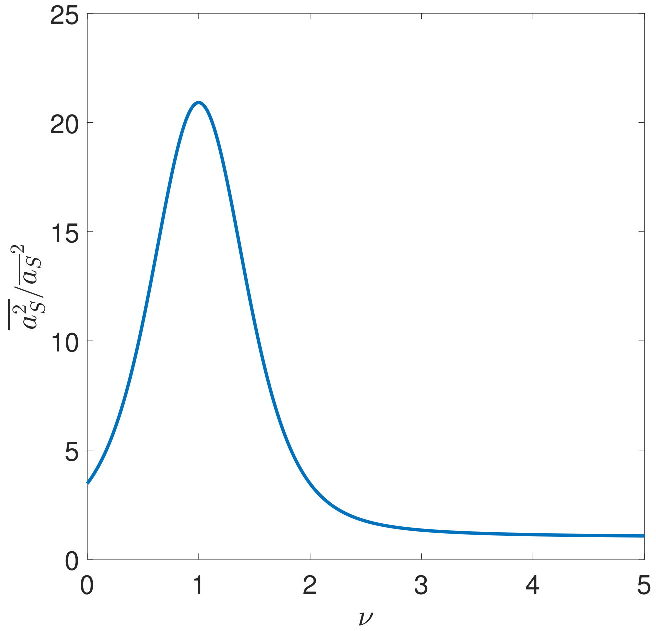

Supplementary Fig. 2: **Heterogeneity of the activity distribution.** We plot the ratio $\overline{a_S^2}/\overline{a_S}^2$ as a function of exponent ν, considering $\rho(a_S, b_S)$ given by Eq. (83) with $a_S \in [a_m, a_M]$, $\eta = a_M/a_m$ and fixing $\eta = 10^3$.

II. SUPPLEMENTARY METHOD 2: CONTINUOUS TIME GILLESPIE-LIKE ALGORITHM FOR NETWORK DYNAMICS AND EPIDEMIC EVOLUTION

The network dynamics is performed by a continuous-time Gillespie-like algorithm [6]: we assign to each node the activity a_S and attractiveness b_S drawn from the joint distribution $\rho(a_S, b_S)$. Initially, the network evolves in the absorbing state, i.e. all nodes are susceptible and infection does not propagate, up to a relaxation time t_0, to reach the equilibrium of activation dynamics:

1. The first activation time, t_i, of each node i is drawn from $\Psi_{a_S^i}(t_i) = a_S^i e^{-a_S^i t_i}$ at time $t = 0$.

2. The node i with the lowest t_i activates and connects m randomly-selected nodes, with probability proportional to their attractiveness b_S.

3. The next activation time t_i for node i is set to $t_i + \tau$, with τ inter-event time drawn from $\Psi_{a_S^i}(\tau)$.

4. All links are destroyed and the process is iterated from point 2.

Then we start the epidemic and contact tracing dynamics as follows:

1. At time $t = t_0$ the population is divided into a configuration of susceptible (S) and infected (P or A) nodes, moreover each node has an activation time $t_i > t_0 = t$ obtained from the initial relaxation dynamics.

2. Node i with the lowest t_i activates. Asymptomatic (A, T and Q) and symptomatic nodes (I) at time t recover at t_i with probability respectively $1 - e^{-\mu(t_i - t)}$ and $1 - e^{-\mu_I(t_i - t)}$: recovered nodes change their activity and attractiveness into $(a_R, b_R) = (a_S, b_S)$. Traced nodes at time t are isolated at t_i with probability $1 - e^{-(t_i - t)/\tau_C}$ (with $\tau_C > 0$ for manual CT and $\tau_C = 0$ for digital CT) and set their activity and attractiveness to zero $(a_Q, b_Q) = (0, 0)$.

3. Pre-symptomatic nodes at time t develop symptoms at t_i with probability $1 - e^{-\gamma_P(t_i - t)}$: they set to zero their activity and attractiveness $(a_I, b_I) = (0, 0)$ and the contact tracing is activated.

   Manual CT: the protocol is enabled for every symptomatic node. Every contact made in the last T_{CT} period has $\varepsilon(a_S)$ probability of being identified and tested, with a_S activity of the symptomatic node. Every node tested and found infected asymptomatic A becomes traced T.

   Digital CT: the protocol is enabled only if the symptomatic node has downloaded the app. Each contact made in the last T_{CT} period with an individual who downloaded the app is identified and tested. Every node tested and found infected asymptomatic A becomes traced T.

4. We set the actual time $t = t_i$ and the active agent i generates exactly m links with m nodes randomly-chosen with probability proportional to their attractiveness b at time t (depending on their status and

isolation). The contacts are registered in the contact list of both nodes engaged in the connection. If the link involves a susceptible and an infected node (P, A or T), a contagion can occur with probability λ and the susceptible node becomes pre-symptomatic with probability δ or asymptomatic with probability $1-\delta$.

5. The new activation time of node i is $t_i = t+\tau$ and it is obtained drawing the inter-event time τ from the inter-event time distribution $\Psi_{a^i}(\tau) = a^i e^{-a^i \tau}$, where a^i is the activity of node i at time t. All the links are deleted and the process is iterated from point 2.

III. SUPPLEMENTARY NOTES: ROBUSTNESS OF THE RESULTS

The advantage of the manual CT is robust relaxing many assumptions and changing most parameters in the modelling scheme.

A. Activity-attractiveness distribution, limited scalability parameters and delays

Supplementary Fig. 3(a) shows the distribution $P(k)$ of the number of contacts made by an individual over a T_{CT} period and the distribution $P(k_T)$ of the number of contacts k_T traced by an index case with manual CT in the presence of limited scalability (recall probability as Eq.(2) of the main paper with $k_c = 130$ [7]), for a realistically heterogeneous population. The average number of contacts traced by an index case is approximately $10-60$ (depending on $\bar{\varepsilon} \sim 0.1-0.5$), consistent with the reported data [7, 8]. Analogous contact patterns are generated by changing k_C or other parameters of the activity distribution.

The results on the comparison between manual and digital CT are robust to changes in the maximum number of traceable contacts k_c in the manual CT: an increase in k_c reduces the effects of limited scalability; similarly, its reduction makes the effects of limited scalability stronger. However, modifying k_c with realistic values changes only slightly the epidemic threshold of the manual contact tracing protocol, without changing qualitatively the results, since the manual protocol remains more effective than the digital one, for small $\bar{\varepsilon} = f^2$ values (Supplementary Fig. 3(b)).

The results are also robust when considering changes in the social properties of the population, i.e. assuming a different functional form of the $\rho(a_S, b_S)$ distribution. For example, we can assume that all nodes feature equal attractiveness b and different activity: $\rho(a_S, b_S) = \rho_S(a_S)\delta(b_S - b)$. In this case the correlations between activity and attractiveness are removed: however, again the manual CT is more effective than the digital one in heterogeneous populations and for small $\bar{\varepsilon} = f^2$ (Supplementary Fig. 3(c)). The differences between the two methods are reduced, compared to the case with correlations (see the inset of Supplementary Fig. 3(c)), due to the reduction in heterogeneities, since homogeneous terms are introduced assuming all the nodes having the same attractiveness. However, differences between the two protocols remain evident, even in the presence of delays $\tau_C > 0$ and limited scalability in the manual CT.

Similarly, the advantage of manual contact tracing holds also considering limited scalability and stronger delays in manual contact tracing τ_C: even for a delay of 7 days the manual CT remains more effective than the digital one, for small $\bar{\varepsilon} = f^2$ and in heterogeneous populations (Supplementary Fig. 3(d)).

B. Epidemic active phase

The results are also robust when considering the effects of contact tracing on the active phase of the epidemic: as expected from the analysis on the epidemic threshold, the differences between the two methods are reduced increasing τ_C, however even with considerable delays τ_C the manual CT for small values of $\bar{\varepsilon} = f^2$ is more effective in flattening the infection peak and in lowering the epidemic final-size (Supplementary Fig. 4). Indeed, its effectiveness, compared to the digital CT, is maximized for $\tau_C = 0$ (Supplementary Fig. 4(a)); the differences are reduced but still present considering strong delays in manual CT, such as $\tau_C = 5$ days (Supplementary Fig. 4(b)). The differences remain even if we consider the system deeply in the active phase, that is for $r \gg r_C$: in this case the differences are sightly reduced due to the high infectivity of the system, however again the manual method for small $\bar{\varepsilon}$ is more effective than digital CT (Supplementary Fig. 4(c)). Finally, as observed for the epidemic threshold, for very large $\bar{\varepsilon} = f^2 = 0.6$ and strong delays $\tau_C = 5$ days, the digital protocol becomes more effective in reducing the impact of the epidemic, further flattening the infection peak and reducing the epidemic final-size (Supplementary Fig. 4(d)). This again confirms that the effects of the protocols on the active phase are similar to those observed on the epidemic threshold, including the differences in the two approaches.

C. Deterministic household CT

Typically when an individual becomes an index case, developing symptoms, her household is always traced and isolated, regardless of the CT protocol implemented. To take into account this effect, we augment both CT

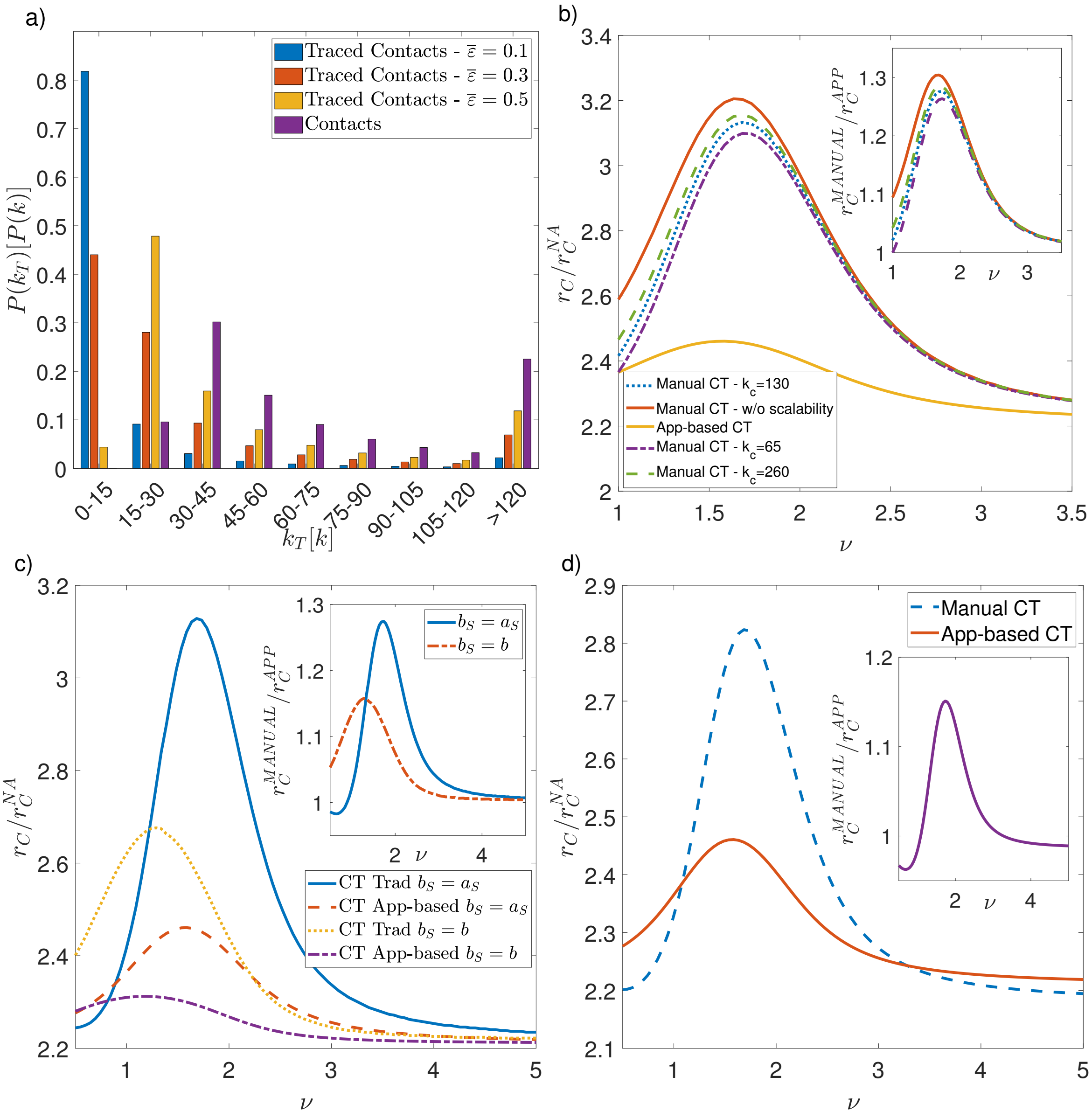

Supplementary Fig. 3: **Robustness of the results for the epidemic threshold.** In panel (a) we plot the distribution $P(k)$ of contacts made by an individual over a T_{CT} period and the distribution $P(k_T)$ of contacts traced by an index case with manual CT in the presence of limited scalability ($k_C = 130$) for fixed $\bar{\varepsilon}$ (see legend) and for $\rho(a_S, b_S)$ given by Eq. (83) with $\nu = 1$. In panels (b)-(d) we plot the ratio between the epidemic threshold r_C in the presence of contact tracing protocols and the epidemic threshold of the non-adaptive case r_C^{NA}. The ratio is plotted for both manual and digital contact tracing, as a function of ν. In all the insets we plot the ratio between the epidemic threshold of the manual contact tracing r_C^{MANUAL} and that of the app-based contact tracing r_C^{APP}, as a function of ν. In panel (b) the ratio for the manual CT is plotted for several values of k_c and also when scalability is not considered (legend), setting $\tau_C = 3$ days and $\rho(a_S, b_S)$ given by Eq. (83). In panel (c) the ratio is plotted both for $\rho(a_S, b_S) \sim a_S^{-(\nu+1)}\delta(b_S - a_S)$ and for $\rho(a_S, b_S) \sim a_S^{-(\nu+1)}\delta(b_S - b)$ (legend), setting $\tau_C = 3$ days and $k_c = 130$. In panel (d) the ratio is plotted for $\rho(a_S, b_S)$ given by Eq. (83) setting $\tau_C = 7$ days and $k_c = 130$. In all panels $a_S \in [a_m, a_M]$ with $a_M/a_m = 10^3$, $\overline{a_S} = 6.7\, days^{-1}$, $T_{CT} = 14$ days, and in panels (b)-(d) $\bar{\varepsilon} = f^2 = 0.1$, $\delta = 0.57$, $\tau_P = 1.5$ days, $\tau = 14$ days.

protocols with a deterministic contribution: a number of contacts, corresponding to the household, is always traced, both in digital and in manual CT. The augmented digital tracing is implemented by means of the hybrid CT formalism, assuming that the index case manually traces at least s contacts (household size) or, if she has had less, traces them all. The recall probability of this manual part is $\varepsilon_h(a_S)$ defined as in Eq. (2) of the main paper, with $k_C = s$ and $\varepsilon_h^* = 1$. The probability of tracing a contact within this augmented digital protocol is $f^2 + \overline{\varepsilon_h}$ (where $\overline{\varepsilon_h} = \int da_S \varepsilon_h(a_S)\rho_S(a_S)$): thus, to compare its efficacy with the manual CT, we fix the same probability to trace a contact, that is $\bar{\varepsilon} = f^2 + \overline{\varepsilon_h}$. In this way, even for the manual CT at least the tracing of the household is necessarily performed.

Supplementary Fig. 5(a)-(c) shows that for low values of $\bar{\varepsilon} = f^2 + \overline{\varepsilon_h}$ (i.e. small f^2) manual CT is more advantageous than the digital one, even taking into account the deterministic tracing of contacts in the household. The digital protocol becomes more effective only for high app adoption rates and unrealistic long delays:

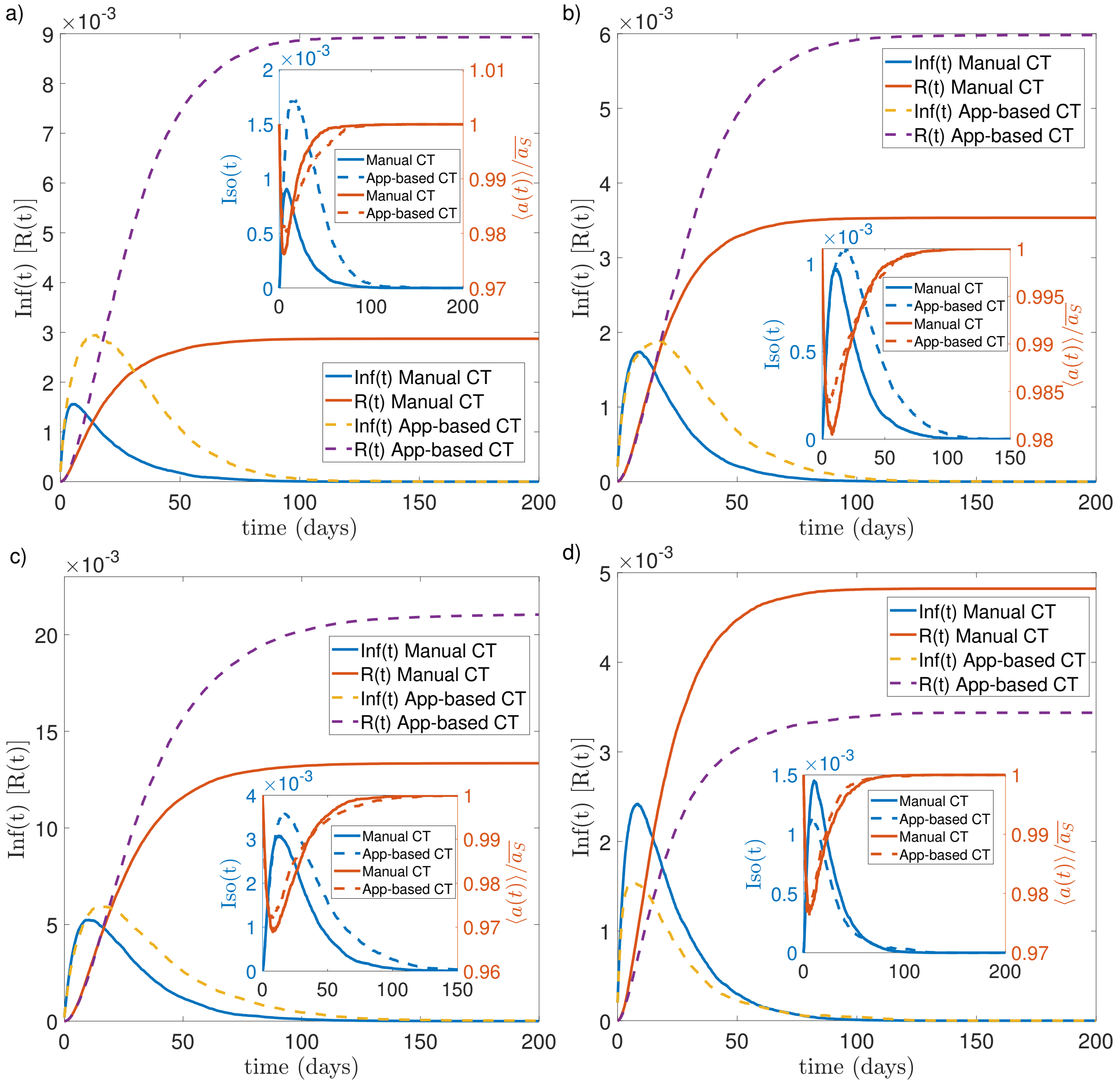

Supplementary Fig. 4: **Robustness of the results for the epidemic active phase.** In all panels we plot the temporal evolution of the fraction of infected nodes $Inf(t)$, i.e. infected asymptomatic and infected symptomatic, and of the fraction of removed nodes $R(t)$, for both manual and digital CT. In the insets we plot the temporal evolution of the fraction of isolated nodes $Iso(t)$ (right y-axis) and of the average activity of the population $\langle a(t)\rangle$ (left y-axis), normalized with $\overline{a_S}$. All curves are averaged on several realization of disorder and temporal evolution. In panel (a) we set $\tau_C = 0$, $\overline{\varepsilon} = f^2 = 0.1$, $r/r_C^{NA} = 4.0$ and the curves for digital and manual CT are averaged respectively over 379 and 445 realizations; in panel (b) we set $\tau_C = 5$ days, $\overline{\varepsilon} = f^2 = 0.1$, $r/r_C^{NA} = 3.1$ and the curves for digital and manual CT are averaged respectively over 554 and 604 realizations; in panel (c) we set $\tau_C = 5$ days, $\overline{\varepsilon} = f^2 = 0.1$, $r/r_C^{NA} = 7.0$ and the curves for digital and manual CT are averaged respectively over 311 and 348 realizations; in panel (d) we set $\tau_C = 5$ days, $\overline{\varepsilon} = f^2 = 0.6$, $r/r_C^{NA} = 4.5$ and the curves for digital and manual CT are averaged respectively over 552 and 469 realizations. In all panels $\rho(a_S, b_S)$ is given by Eq. (83) with $a_S \in [a_m, a_M]$, $a_M/a_m = 10^3$, $\nu = 1.5$, $\overline{a_S} = 6.7\, days^{-1}$, $N = 5\,10^3$, $\delta = 0.57$, $\tau_P = 1.5$ days, $\tau = 14$ days, $k_c = 130$, $T_{CT} = 14$ days and the errors, evaluated through the standard deviation, are smaller or comparable with the curves thickness.

therefore, our results are robust to the addition of this deterministic household CT contribution.

D. Correlation between probability of app adoption and individual activity

We considered the probability of downloading the app uniform over the population, since economic and personal factors can produce opposite forces which correlate and anticorrelate the probability of downloading the app with the individuals' activity. Moreover personal data of CT app users are not available, due to privacy issues. However, evidences are currently emerging that those who download the app are individuals who engage very cautious behaviors, i.e. f and a_S are anticorrelated [9, 10].

We implement the hybrid CT protocol on a heterogeneous population, investigating the effectiveness of digital CT varying the app adoption level $\overline{f} = \int da_S f(a_S)\rho_S(a_S)$ and the correlations between f and a_S. We consider

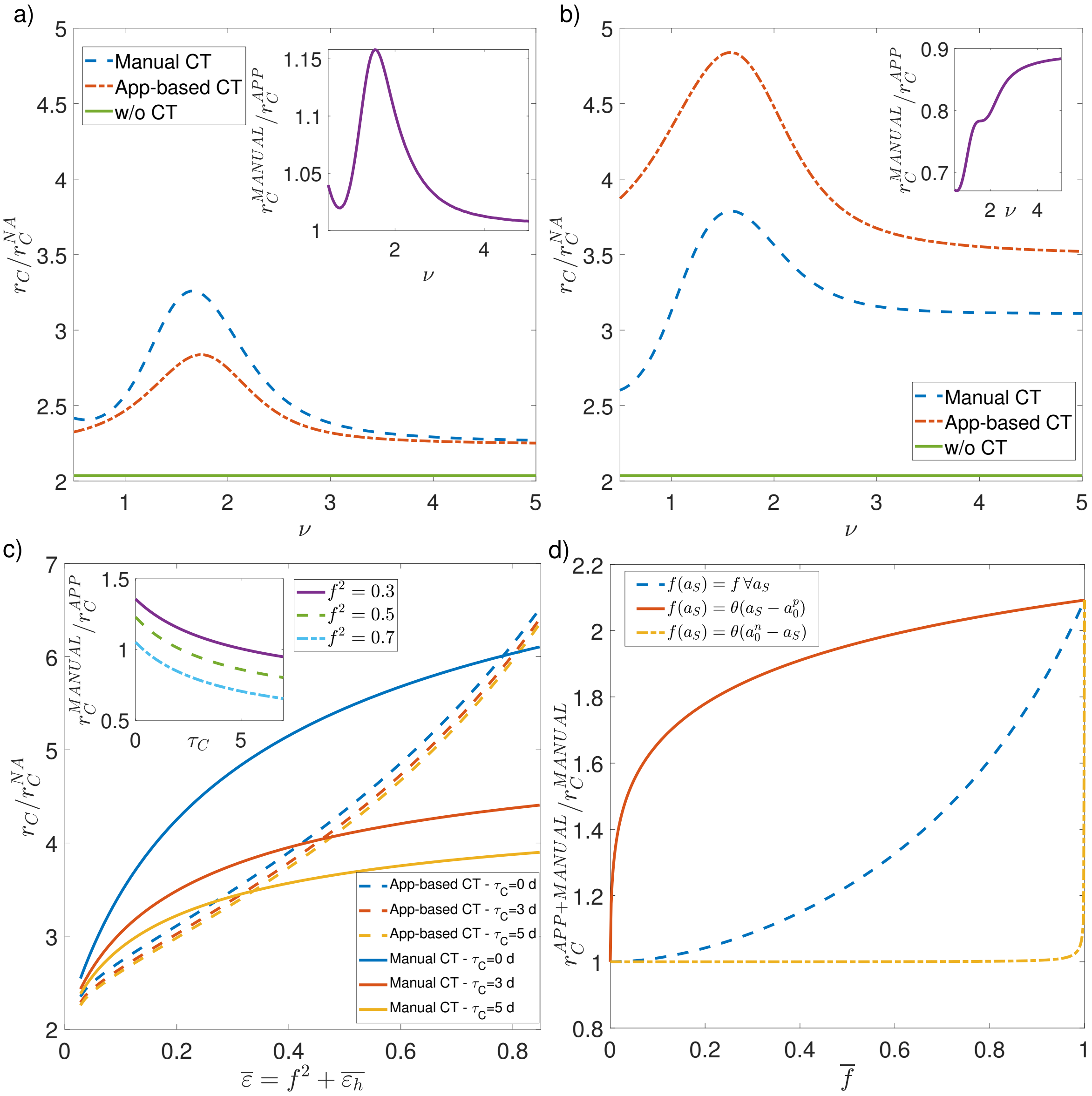

Supplementary Fig. 5: **Effects of deterministic household contact tracing and effects of correlations between app adoption and activity.** In panels (a) and (b) we plot, as a function of the exponent ν, the ratio between the epidemic threshold r_C for both CT protocols and the epidemic threshold of the non-adaptive case r_C^{NA}, with limited scalability, delay in manual CT and the additional deterministic household CT. In the insets we plot the ratio between the epidemic threshold of the augmented manual CT, r_C^{MANUAL}, and that of the augmented app-based CT, r_C^{APP}, as a function of ν. In panel (a) $f^2 = 0.1$ and $\tau_C = 3$ days, while in panel (b) $f^2 = 0.6$ and $\tau_C = 5$ days. In panel (c) we plot the ratio r_C/r_C^{NA} as a function of $\overline{\varepsilon} = f^2 + \overline{\varepsilon_h}$ for both augmented CT protocols, setting $\nu = 1.5$ and for several values of τ_C: in the inset we plot the ratio r_C^{MANUAL}/r_C^{APP} as a function of τ_C for several f^2 values. In all panels (a)-(c) we fix $s = 3$ (size of household) and $\varepsilon_h^* = 1$, corresponding to $\overline{\varepsilon_h} = 0.028$. In panel (d) we plot, as a function of $\overline{f}$, the ratio between $r_C^{MANUAL+APP}$, the epidemic threshold when the hybrid CT protocol is implemented, and r_C^{MANUAL}, the epidemic threshold when only the manual CT is implemented. The curves correspond to the uncorrelated, positively correlated and negatively correlated $f(a_S)$ (see legend), and are obtained fixing $\overline{\varepsilon} = 0.4$, $\tau_C = 3$ days, $\nu = 1.5$. In all panels the distribution $\rho(a_S, b_S)$ is given by Eq. (83), $a_M/a_m = 10^3$, $\overline{a_S} = 6.7\,days^{-1}$, $\delta = 0.57$, $\tau_P = 1.5$ days, $\tau = 14$ days, $k_c = 130$, $T_{CT} = 14$ days.

three extreme cases of correlations: the uncorrelated case in which $f(a_S) = \overline{f} = f\ \forall a_S$; the completely positively correlated case $f(a_S) = \theta(a_S - a_0^p)$, with $\theta(x)$ the Heaviside step function; the completely negatively correlated case $f(a_S) = \theta(a_0^n - a_S)$. These borderline cases are hardly realistic, however they allow to obtain useful information on the role of correlations: more realistic shapes of $f(a_S)$ are interesting directions for future work.

To compare these three cases, we set the thresholds a_0^n and a_0^p by fixing $\overline{f}$: Supplementary Fig. 5(d) shows that correlations have a strong impact on the effectiveness of digital CT. If the app is downloaded from all hubs (positive correlations) a low level of adoption $\overline{f} \sim 0$ is enough to obtain a significant increase in the epidemic threshold; on the contrary, if the app is downloaded only by very cautious people (negative correlations) the effect of the digital CT becomes significant only for very high adoption level $\overline{f} \sim 1$, worse than the uncorrelated case.

The effects of correlations in app adoption further strengthens our results, highlighting the dominant role of

manual CT in the current situation of negative correlations [9, 10]. Furthermore these results suggest future directions to make digital CT more effective exploiting heterogeneities.

Supplementary References

[1] Mancastroppa, M., Vezzani, A., Muñoz, M. A. & Burioni, R. Burstiness in activity-driven networks and the epidemic threshold. *J. Stat. Mech.: Theory Exp* **2019**, 053502 (2019).
[2] Tizzani, M. *et al.* Epidemic spreading and aging in temporal networks with memory. *Phys. Rev. E* **98**, 062315 (2018).
[3] Pozzana, I., Sun, K. & Perra, N. Epidemic spreading on activity-driven networks with attractiveness. *Phys. Rev. E* **96**, 042310 (2017).
[4] Ghoshal, G. & Holme, P. Attractiveness and activity in internet communities. *Physica A* **364**, 603 – 609 (2006).
[5] Mancastroppa, M., Burioni, R., Colizza, V. & Vezzani, A. Active and inactive quarantine in epidemic spreading on adaptive activity-driven networks. *Phys. Rev. E* **102**, 020301 (2020).
[6] Gillespie, D. T. A general method for numerically simulating the stochastic time evolution of coupled chemical reactions. *J. Comput. Phys.* **22**, 403 – 434 (1976).
[7] Keeling, M. J., Hollingsworth, T. D. & Read, J. M. Efficacy of contact tracing for the containment of the 2019 novel coronavirus (covid-19). *J. Epidemiology Community Health* **74**, 861–866 (2020).
[8] Visontay, E. Victoria's contact-tracing effort buckles under the weight of covid-19 cases. *The Guardian* (2020). 08-04-2020.
[9] Wyl, V. v. *et al.* Drivers of acceptance of covid-19 proximity tracing apps in switzerland. Preprint at https://www.medrxiv.org/content/10.1101/2020.08.29.20184382v2.full (2020)
[10] Saw, Y. E., Tan, E. Y., Liu, J. S. & Liu, J. C. Predicting public take-up of digital contact tracing during the covid-19 crisis: Results of a national survey. Preprint at https://www.medrxiv.org/content/10.1101/2020.08.26.20182386v2 (2020)
